# Adaptive Printing of Conductive Microfibers for Seamless Functional Enhancement Across Diverse Surfaces and Shapes

**DOI:** 10.1007/s42765-025-00561-6

**Published:** 2025-05-15

**Authors:** Stanley Gong Sheng Ka, Wenyu Wang, Henry Giddens, Zhuo Chen, Ahsan Noor Khan, Yuan Shui, Andre Sarker Andy, Shuyu Lyu, Tawfique Hasan, Yang Hao, Yan Yan Shery Huang

**Affiliations:** 1 Department of Engineering, University of Cambridge, Trumpington Street, Cambridge, CB2 1PZ UK; 2 The Nanoscience Centre, University of Cambridge, 11 JJ Thomson Avenue, Cambridge, CB3 0FF UK; 3 Cambridge Graphene Centre, University of Cambridge, 9 JJ Thomson Ave., Cambridge, CB3 0FA UK; 4 Thrust of Smart Manufacturing, Hong Kong University of Science and Technology (Guangzhou), Guangzhou, China; 5 School of Electronic Engineering and Computer Science, Queen Mary University of London, 10 Godward Square, London, E1 4FZ UK

**Keywords:** Fiber, Sensor, Functionalization, Transient electronics, Customization, Fiber-of-things (FoT)

## Abstract

Developing methods to non-destructively deposit conductive materials onto existing objects can enhance their functionalities on-demand. However, designing and creating such structures to accommodate diverse shapes and surface textures of pre-fabricated objects remains challenging. We report an on-demand printing strategy for creating substrate-less, conducting microfiber patterns that can be adaptively deposited onto a wide range of objects, including daily-use stationery, tools, smartwatches, and unconventional materials like porous graphene aerogels. Solution-drawn microfibers are directly deposited onto the object in a semi-wet state upon synthesis, enabling seamless fiber-object integration in a single step. The design and format of the microfiber patterns can be tuned on-demand to adapt to the shapes and surface textures of target objects, ensuring compatibility with user-specific applications. These air-permissive, highly transparent layers minimally obstruct the original appearance and functions of the objects while equipping them with additional sensing, energy conversion, and electronic connectivity capabilities.

**Graphical abstract:**

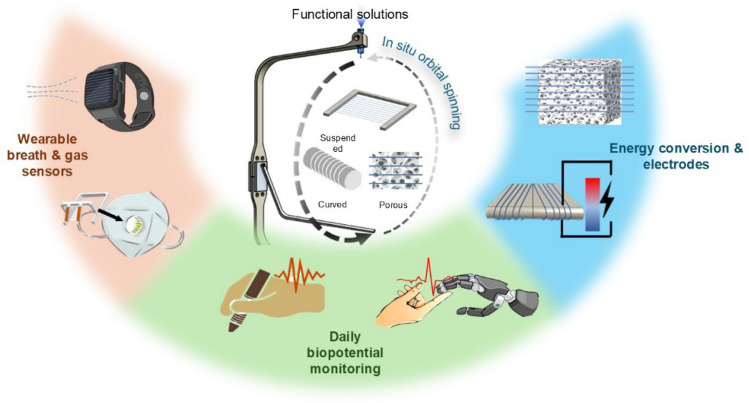

**Supplementary Information:**

The online version contains supplementary material available at 10.1007/s42765-025-00561-6.

## Introduction

Enhancing the sensing and electronic connection capabilities of existing objects could additionally extend their applications and servicing life. For example, conducting fibrous networks when attached onto a face mask, could enable the face mask with breath sensing function [1, 2]; film-based sensing layers could be applied onto the fingers of robotic hands, enabling them with biomimetic tactile perceptions [3, 4]. Without the need to replace the existing objects, attaching conducting material layers onto their surfaces could be a simple and cost-effective solution to extend their utilities [5–7]. To provide such add-on functions in a timely manner and to satisfy the fast-changing demands of the users, it is important to achieve on-demand design, fabrication, and deployment of the conducting material layers at the point of use.

Challenges remain in creating and depositing the conducting material layers on-demand to adapt with the diverse shape and surface textures of the base objects, while minimally obstructing the original appearance and functions of the base objects. While three-dimensional (3D) printing could produce customized conductive tracks [8, 9], printing electronic circuits in situ on existing objects often requires vision-assisted path planning for accurate material deposition. Therefore, print resolution and precision could be compromised by the presence of the surface textures. Spraying functional inks onto base objects is a straightforward method of adding extra functionalization [10], yet this approach can be crude and cause undesirable changes to the appearance of the original base objects. Furthermore, successful deployment of functional inks via spraying is highly dependent on the target surface’s morphology and the ink’s wetting behavior, which could limit the applicability of this approach.

Recent advances have opened the possibilities for synthesizing highly conducting microfibers for transient functionalization [11–16]. Owing to their nano- to micro-scaled diameter, the microfiber-based structures are flexible, transparent, and gas-permeable [17, 18]. Thus, compared to thick films or 3D printed structures, using conducting microfibers as the add-on functionalization layers could potentially minimize the influence on the original appearance and function of the base objects. However, current nano- and microfiber-based structures are usually fabricated ex situ, hence requiring additional transfer processes from the deposition site to the target surface. Besides, the microfiber layer would further require an additional adhesive layer in the device system to be deployed and adhered to the target surfaces [19–21]. With these complicated two-step fabrication and transfer processes, the microfibers may not adapt to various shapes, surface textures, and sizes of intended base objects, especially objects with large curvature or porous surfaces.

Herein, we present a one-step adaptive fiber deposition strategy to achieve on-demand creation and adaptive deposition of conducting microfiber patterns with tunable design features to suit with the base objects and intended utility enhancement scenarios. With the in situ solution spinning strategy, the as-synthesized microfibers would deposit on the targeted surfaces in a semi-liquid state, and hence enabling direct and conformal adhesion before solidification. This fabrication process enables the conducting microfibers to adapt with various base objects of different shapes, geometries, and material surface textures [22], from everyday items such as stationery, face masks, and smartwatches to unconventional materials like highly porous graphene aerogels, hence enabling sensing, energy conversion, and electronic connections at the point of use. The microfibers’ small diameter, absence of a substrate, and extensive pattern openness ensure high permissiveness and optical transparency. In addition, the conducting microfibers are permissive to electromagnetic (EM) waves in the gigahertz range; this feature ensures the conducting microfiber patterns minimally impede signal transmission and wireless communications when deposited onto electronic devices such as smartwatches and near-field communication (NFC) tags. By tailoring the formats and materials of the conducting microfiber patterns, we show on-demand utility enhancement of daily objects with health monitoring and energy conversion functions; and we show the conducting microfibers could be used to connect highly porous graphene aerogels without shielding their porosity for gas sensing. Such target-adaptive microfiber functionalization strategy provides a flexible and efficient approach for integrating customizable electronic functions onto various existing objects, enhancing internet-of-things connectivity and showing potentials for a sustainable “Fiber-of-Things” (FoT) future.

## Experimental Section

### Fiber Solution Preparation

Fiber solutions were prepared by mixing the functional inks (poly(3,4-ethylenedioxythiophene):polystyrene sulfonate (PEDOT:PSS) solution or silver ink) with the base polymer matrix solution. The base polymer matrix and PEDOT:PSS solutions were prepared according to previous protocols [23]. The silver ink was prepared using modified Tollen’s process [24]. In brief, 2.5 ml of ammonium hydroxide (28%, Alfa Aesar) and 200 μL of formic acid (Thermo Fisher Scientific) were added into 1 g of silver acetate (Sigma-Aldrich) powder. The solution was stirred for 2 h at room temperature to obtain a transparent liquid, followed by filtration with a 0.22 μm PTFE (polytetrafluoroethylene) filter (Gilson Scientific). The final fiber solution for PEDOT:PSS or silver nanoparticles (AgNP) fibers was prepared by mixing either the PEDOT:PSS ink or silver ink with the base polymer matrix solution in 1.5–1 volume ratio respectively, followed by stirring at room temperature for 12 h.

### Aerogel Preparation

The aerogel was prepared by synthesizing the SnO_2_/GO hybrid materials following the surfactant-assisted hydrothermal growth process. First, graphene oxide (GO) dispersion in water is prepared by dispersing non-exfoliated GO paste (Sigma-Aldrich) in deionized (DI) water with a concentration of 25 mg mL^−1^, followed by rigorous mechanical stirring for 3 h. SnO_2_ precursor is separately prepared by dissolving 4 mmol of tin chloride pentahydrate (SnCl_4_·5H_2_O, Sigma-Aldrich) and 4 mmol 6-aminohexanoic acid (AHA, Sigma-Aldrich) in 30 mL DI water and sonicating the solution for 5 min. This precursor is subsequently mixed with 10 mL GO dispersion by sonication for 30 min and transferred to a PTFE-lined digestion vessel (4744, Parr Instrument) for hydrothermal growth at 140 °C for 3 h. The vessel is cooled down to room temperature in cold water, and the product is centrifuged at 4000 rpm and washed with DI water 3 times before being re-dispersed in water with a concentration of 15 mg mL^−1^. Finally, 50 mmol/L of copper chloride (CuCl_2_, Sigma-Aldrich) and 28 mmol/L of ascorbic acid (AA, Acros Organics) are added into the as-synthesized suspension for crosslinking and gelation. The mixture is heated at 60 °C for 1 h in the oven and used within one day as ink for 3D printing.

The SnO_2_/GO aerogel gas sensors are fabricated with a custom-built 3D printing setup. The hybrid ink is first loaded into a 5-mL syringe connected with a dispensing tip, then extruded at a constant flow rate and printed out into 5 mm × 5 mm × 2 mm blocks. The aerogel blocks are then frozen in liquid nitrogen for 10 min and transferred to a freeze dryer (LyoQuest, Telstar) for overnight freeze-drying. The resulting aerogels are soaked in 100 mmol/L of copper chloride (CuCl_2_, Sigma-Aldrich) solution in ethanol for 1 h to introduce additional metal doping through liquid-phase ligand exchange. The samples are washed twice with hexane, dried in air and then annealed at 160 °C for 3 h to further reduce GO before being ready for subsequent fiber spinning process and gas sensing measurements.

### Microfiber Deposition

The microfibers were printing following the in situ orbital spinning approach [23]. In brief, solutions were loaded into a 1-ml plastic syringe which was connected to a 22-gauge blunt-end stainless steel needle. Compressed air was used to feed the solution via an air flow controller (Elveflow). The air pressure was maintained at around 70 mbar for PEDOT:PSS fiber solution and around 20 mbar for AgNP fiber solution. For AgNP fibers, post-calcination (100 °C for 15 min on a heating plate in room conditions, RS Component) was needed in order to reduce the silver compound into elemental silver. The PEDOT:PSS fibers could be used directly after printing.

### Fiber Characterizations

The shear rheological properties of various fiber solutions were acquired with a Kinexus KNX2112 controlled stress rheometer at 25 °C. A parallel plate configuration was used with a 1 mm gap distance.

The SEM (scanning electron microscopy) images and EDS (energy dispersive X-ray spectroscopy) analysis of the fibers were acquired with FEI Philips Dualbeam Quanta 3D SEM equipment. The cyclic voltammetry measurements were acquired by a PalmSens 4 potentiostat with an array of around 500 fibers deposited on a glass slide with the array length *L* = 50 mm and array width *d* ~ 25 mm. The electromechanical properties of the fiber arrays were measured with a self-assembled tensile rig, the assembly of the tensile rig was previously reported [25]. An array of around 400 suspended fibers with the array length *L* = 5 mm and array width *d* ~ 20 mm was pulled from the middle, and the resistance of the fiber array was monitored by a multimeter (34465A, Keysight Technologies). In the bending test, the same suspended fiber array was printed onto a self-designed bending rig where the gap between two electrical contact pads could change. The original gap distance between the two electrical contact pads was 5 mm (or *L*_*0*_ = 5 mm), and the gap was contracted to 2 mm (or *L*′ = 2 mm) until being retracted back for each cycle. The cycle frequency was set at 1 Hz. The visible light transmittance spectra was acquired through JENWAY 7250 UV/Vis Spectrophotometer. The samples were prepared by depositing an array of around 300 fibers with array length *L* = 10 mm and array width *d* ~ 20 mm onto microscope slides.

The microwave frequency transmission behavior through the samples was investigated through S-Parameter measurements at two frequency bands. At the low GHz band covering 1–3 GHz, two quarter wavelength monopole antennas were positioned either side of a PEC reflector with a window cut in the center that allowed weak coupling between the two elements. The sample was placed over the window and the two-port S-Parameters were measured on an Anritsu MS46322A Vector Network Analyzer. At the 26–40 GHz band, the samples were positioned between two WR28 waveguide sections and the S-Parameters were measured using a Rhode & Schwarz ZNA-43 Network Analyzer. The results are shown as S21 parameter—the ratio of transmitted to reflected power between the two ports. In all cases, a measurement of free space path loss in place of the sample was taken for reference. Substrate-less fiber networks were printed on rectangular 3D printed polylactic acid (PLA) frames of 5 cm × 5 cm sizes. The fiber network was composed of two perpendicular fiber arrays in both horizontal and vertical directions, in each direction, the fiber array was composed of around 1000 fibers with both array length and width of around 5 cm. The plastic frame with fibers was then fitted on a 25 cm × 25 cm stainless steel frame before the measurement, in order to shield EM waves bypassing the frame.

### Dual-Design Moisture Flow Sensor

Suspended PEDOT:PSS fibers were first printed onto several of the U-shape plastic blocks (for each block, around 300 fibers were deposited with the array *L* = 25 mm and array width *d* ~ 30 mm). The plastic block was printed with a 3D printer (Ultimaker S3) using polylactic acid (PLA) filament. The copper tapes were then adhered to the two legs of the block prior to fiber printing. Five fiber blocks could then be assembled into either a multi-layer design or a box design. In the multi-layer design, a 3D printed support structure at the back was used. The five blocks were independently connected to an electric circuit programmed by Arduino Uno to detect the real-time fiber array resistances. A diffuser (VicTsing) was used to release water vapor pulses that would pass through the fiber arrays, and the change in resistances respective to the corresponding layers were recorded by a multimeter (34465A, Keysight Technologies).

### Wearable Sensors

For the wearable breath sensors attached to the face mask or the smart watch, a 3D printed U-shape block was designed with locking features to help clamp onto the valve of the face mask (3 M 9501 V), or the screen of the smart watch (Apple Watch 2). Suspended PEDOT:PSS fiber arrays (~ 200 fibers across a distance of 10 mm) were printed across the two legs of the U-shape blocks. The resistance of the fiber array was measured with a multimeter (34465A, Keysight Technologies) during breathing. For gas sensing with smart watches, an array of ~ 300 PEDOT:PSS fibers across 15 mm was printed directly onto the screen of an Apple Watch, with both ends connected to copper tapes. Water mist (from VicTsing aroma diffuser) or ammonia vapor (from a bottle of 2.8% ammonia solution) were applied to the screen of the Apple Watch, while the resistance change of the fiber array was recorded by the multimeter. Human participant experiments were performed with the approval of the Ethics Committee of the Department of Engineering at the University of Cambridge (7/7/2021, CUEDREC) and the informed consent from volunteers.

### Biopotential Measurements with a Robotic Hand and a Plier

To detect the ECG (electrocardiogram) signals with a robotic hand (Motorized Robot Hand Building Set, 4 M Store), a strip of copper tape was first attached to the bottom side of the index finger of the robotic hand to serve as connection to outer circuits. Then, an array of PEDOT:PSS fibers were printed to wrap around the robotic finger (around 400 fibers in the array with array length *L* ~ 10 mm and width *d* ~ 20 mm). During measurement, the index finger of the volunteer was in touch with the fiber-decorated area on the robotic finger. Similarly, an array of around 600 PEDOT:PSS fibers with the array length *L* ~ 10 mm and width *d* ~ 50 mm could be printed to wrap around a pencil, with a strip of copper tape. To detect the ECG and EMG signals from the thumb-tendon area when using a plier (Jonard Industries), a strip of copper tape was placed onto the bottom side of the plier handle, followed by printing an array of around 1000 PEDOT:PSS fibers with the array length *L* ~ 20 mm and width *d* ~ 50 mm. Note the copper tape in all above examples only served as connections between the fibers and the outer circuit, and was not directly in contact with the skin. A hydrogel electrode (H124SG Covidien) was attached to the other hand of the volunteer as counter electrode. The surface impedance was measured with a potentiostat (Palmsens 4), and biopotential signals were measured with an Intan RHS stimulation/ recording controller (128 channels, Intan Technologies). During EMG measurements, the participant was requested to cut a rigid object with different levels of forces, which was estimated with a hand grip exerciser (GaoBangM by Amazon).

### AgNP Fiber Microheater

An array of around 200 AgNP fibers with the array length *L* = 50 mm and width *d* ~ 50 mm was printed onto an NFC tag (NXP Chip NTAG213, 144 Bytes Memory, Round Antenna 45 mm) that was adhered on a glass slide ($\theta$ ~ 35$^\circ$). For voltage–current relationship measurements, both ends of the fiber arrays were connected in series with a multimeter (34465A, Keysight Technologies) for current measurement and a power supply (IPS 2303S, RS PRO). An infrared-ray camera (Teledyne FLIR) was used to estimate the surface temperature of the glass slide. An iPhone 11 was used to detect the NFC tag (NTAG213, NXP Chip).

### Thermoelectric Functionalization

Copper tapes were attached to both sides of a reversible leatherette coaster (B&M Retail) and an array of around 2000 PEDOT:PSS fibers printed to wrap around the coaster with the fiber array length *L* ~ 40 mm and width *d* ~ 100 mm. A glass container (Pyrex) filled with hot water of around 80 °C was placed onto the coaster, and a multimeter (34465A, Keysight Technologies) was used to measure current and voltage output.

### Gas Sensing with Graphene-Based Aerogel

The graphene-based aerogels were prepared as mentioned in Sect. 2.2 from a previously reported protocol [26]. A 3D printed plastic frame was produced and the bottom layer PEDOT:PSS fibers (around 250 fibers in the array with the array length *L* = 5 mm and width *d* ~ 3 mm) was printed on the frame, followed by transferring the aerogel block onto the bottom layer fiber. Then, a top layer PEDOT:PSS fibers of the same configuration was printed in orthogonal directions to the bottom layer fibers. The aerogel was then “sandwiched” in between the two layers of the fibers and connected separately via copper tapes. The gas sensing measurements were carried out in a Kenosistec gas characterization system. Two mass flow controllers (MFC) were used, with one regulating the gas flow of dry air as the balance gas and the other one regulating 1 ppm formaldehyde (CH_2_O) as the target gas. The total gas flow rate was maintained at 500 sccm. Sensor resistance was recorded once every second at a fixed reading voltage, and the measurement chamber environment was maintained at 25 °C and 1 atm. A standardized conditioning process was carried out before each measurement by purging with dry air for 120 min.

## Results and Discussion

### Conducting Microfibers by Adaptive Fiber Deposition

**Fig. 1:**
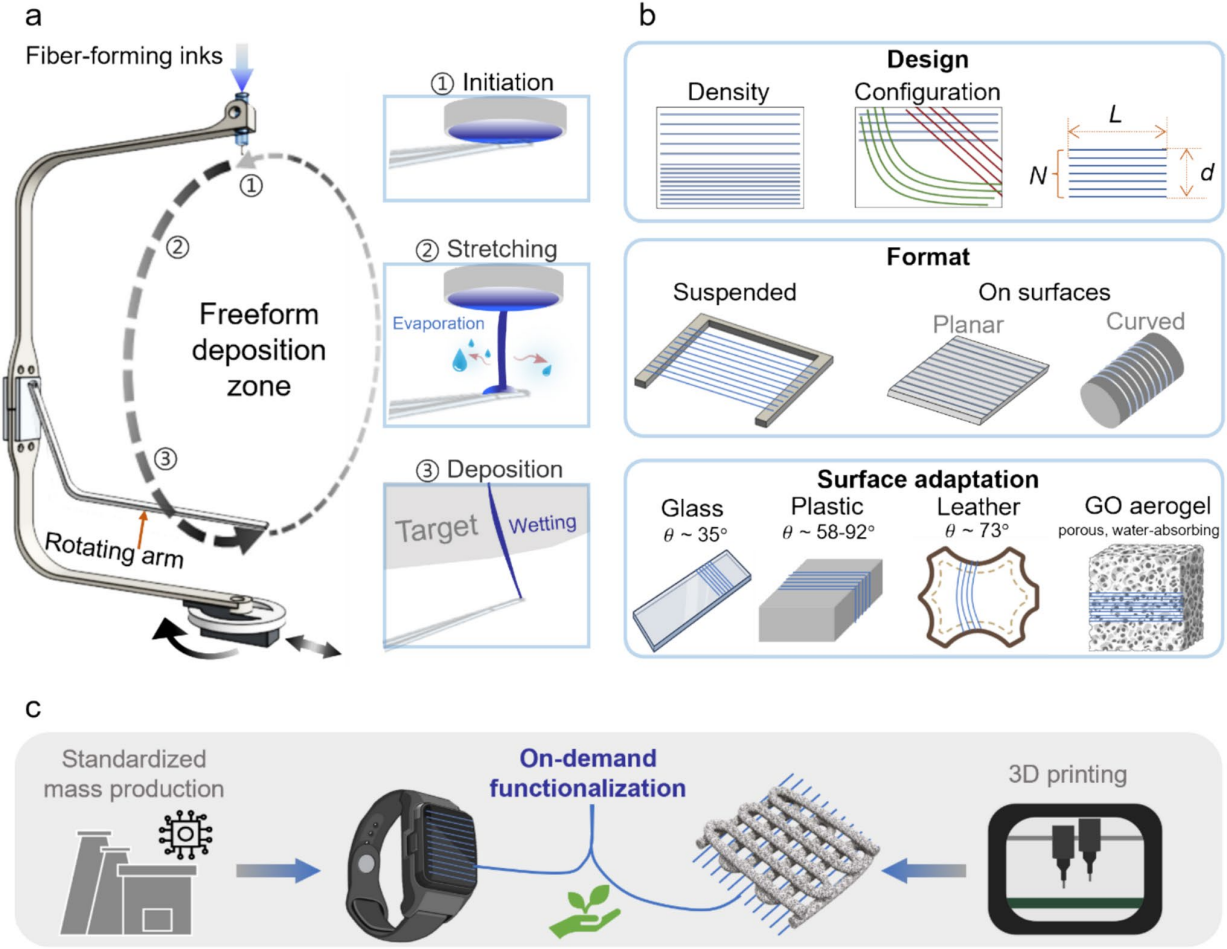
Adaptive fiber deposition. **a** Schematic illustrations showing process of fiber deposition enabled by an orbital spinning set-up, where the microfiber is initiated by a rotating arm to deposit onto a target object placed inside the deposition zone. The aqueous solvent evaporates from the fiber jet during the stretching process. Upon deposition, the fiber jet wetting enables conformity and adhesion to the targeted surface. **b** Adaptive fiber deposition could enable designable fiber patterns and formats, and the fibers are compatible with objects of different shapes and material surface textures ($\theta$ is the water contact angle of these surfaces measured in Fig. S3). **c** The microfibers are in situ deposited to interface with standardized mass-produced objects or 3D printed architecture, providing fit-for-purpose customization with minimized material and energy usage

Adaptive fiber deposition, powered by a previously reported orbital spinning method [23], deposits microfiber patterns directly onto the targeted base objects placed inside the spinning zone (Fig. 1a). In this setup, viscoelastic aqueous fiber-forming inks are fed into a syringe to form a pendent drop on the outlet of a needle. A rotating arm would scratch the pendent drop to initiate an aqueous fiber jet at each rotating cycle. The fiber jet is then stretched and narrowed from the needle outlet to the tip of the rotating arm, eventually landing on the surface of the target object. From fiber jet initiation to surface deposition, the overall process takes around 1–2 s. Thus, upon surface deposition, the fiber jet could remain semi-wet, enabling convenient surface adhesion without the need of specific adhesive material layers or external pressing forces, which are typically required for attaching pre-fabricated dry microfiber-based meshes or devices [19–21]. The fiber wetting could also allow conformity to surfaces with larger curvatures [23]. The adaptive fiber deposition enables versatile microfiber pattern designs and formats while ensuring wide surface adaptations (Fig. 1b).

The orbital spinning mechanism could be mounted to a stage with translational and rotational movement abilities. The programmed movements of the stage could result in designable microfiber patterns with varying densities and orientations (Figure S1). When using the conducting microfibers as the functionalization layer, the pattern design is important for controlling their appearance and electronic characteristics. In a fiber array, the array length (*L*), array width (*d*), and the number of fibers (*N*) are three major design parameters. For the same fiber material and diameter, the directional resistance (*R*) of a parallel fiber array is proportional to the ratio of the array length over number of fibers, or *L/N*; and the ratio of number of fibers over array width, or fiber number density *N/d*, could affect the fiber array transparency (Figure S2 and Supplementary Table 1). Understanding the effects of design parameters on the microfiber patterns could allow appropriate tuning of the appearance and functional characteristics of the functional microfiber layers. Based on the properties of the targeted objects and the intended applications, the microfiber patterns could be designed and printed on-demand. Supplementary Table 1 summarizes the microfiber pattern design parameters adopted for various application scenarios, and Supplementary Table 2 illustrates the versatility of surface and shape adaptation of the on-demand printed microfibers in comparison to other recent fiber functionlization technologies.

The microfibers could either be created in a substrate-less format or printed onto targeted base objects with planar or curved shapes. Although the fiber jet remains semi-wet upon surface deposition, the viscoelasticity of the ink solutions would help to maintain the integrity and continuity of the fibers. Therefore, the microfibers are compatible with a variety of surfaces of different hydrophilicities, ranging from fabrics to plastics, glass, and artificial leather (the water contact angle $\theta$ of these surfaces ranges from 35° to 92°, Figure S3). The microfibers could also be directly deposited onto highly porous surfaces such as graphene aerogels.

The flexible format design and wide surface adaptation of the microfibers could facilitate on-demand integration with a wide range of base objects regardless of their geometries and materials (Fig. 1c). The customizability of the fiber patterns could supplement the high standardization of mass production; and the micrometer-scale feature of the fibers could complement 3D printed structures, which normally have resolutions of hundreds of micrometers [27]. In addition, the adaptive fiber deposition requires minimized material and energy usage and waste generation, providing a sustainable and cost-effective functionalization strategy. The energy used during the adaptive fiber deposition is less than 50 W, much lower than those used in microfabrication [28] and 3D printing [29]; and the material usage and waste generation could be negligible, as estimated to be at a range of ~ 0.01–0.4 mg per device (Supplementary Note 1).

## Opto-electro-mechanical Characterizations of Microfibers

**Fig. 2:**
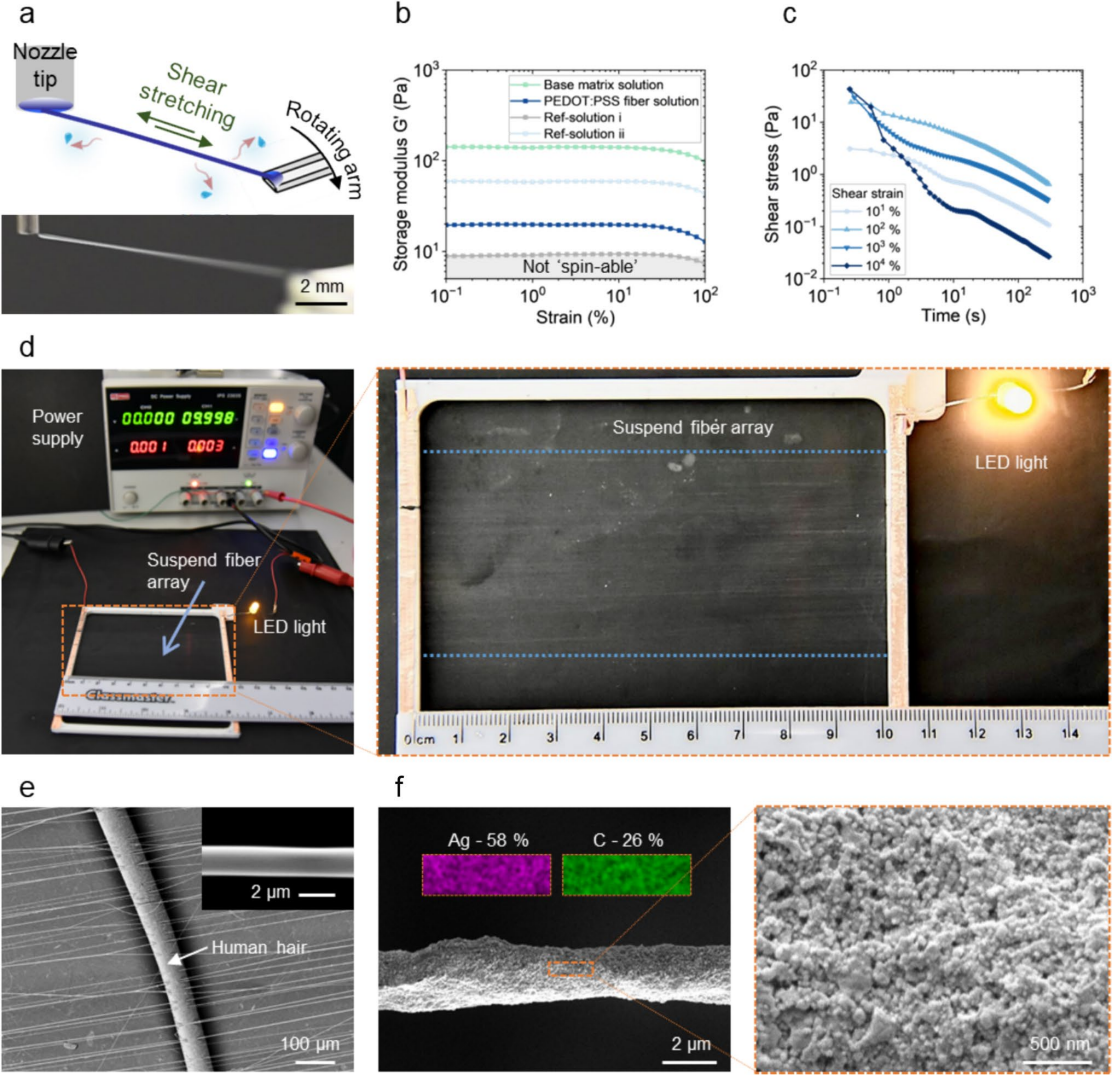
The formation and morphology of microfibers by orbital spinning. **a** Schematic and photo illustrations of the solution fiber initiation process. **b** Solution storage modulus characterized by amplitude sweep experiment at 1 Hz (Ref-solution i and ii are 2 wt% PEO solutions in water of 4 mol/L Da and 8 mol/L Da molecular weight, respectively, and the base matrix solution contains 2 wt% 8 mol/L Da PEO and 0.5 wt% HA in water). **c** Relaxation characterizations of the PEDOT:PSS fiber inks under different shear strains. **d** Photos showing an array of 10 cm length substrate-less PEDOT:PSS fibers used to connect an LED light with the power supply (around 1000 fibers with the array length *L* = 10 cm and width *d* ~ 5 cm). **e** An SEM image showing the PEDOT:PSS fibers in comparison to a human hair, and the insert shows an individual PEDOT:PSS fiber. **f** An SEM image showing the morphology of an AgNP fiber, with insert of EDX analysis showing the carbon and silver contents, and the zoom-in SEM image shows the silver nanoparticles

In the orbital spinning process [23], the fibers are formed from the viscoelastic aqueous ink solutions by shear stretching without the need of additional applied voltages (Fig. 2a). The aqueous solvent contained in the ink continuously evaporates during the fiber jet initiation and surface deposition processes, resulting in the as-deposited fibers remaining in semi-wet status (Figure S4). The molecular chains within the solution need to exhibit long-range and weakly percolated status for the fiber jet to be successfully initiated and maintained between the rotating arm and the nozzle tip. In this work, conducting ink, either PEDOT:PSS ink or silver ink [24], was mixed with a base polymer matrix solution that mainly provides the ‘spin-ability’. The base polymer matrix solution, containing polyethylene oxides (PEO) and hyaluronic acid (HA), was prepared based on a previously reported work [23]. As shown in Fig. 2b, using higher molecular weight PEO and adding HA could increase the elastic modulus of the solution and thus, enhancing the overall ‘spin-ability’. The solution relaxation characterization in Fig. 2c shows that the shear stress required to stretch the fiber jet would not increase when the strain is over 100%. This indicates that the formation of long fibers would not require increasing shear stress input. The viscoelasticity of the solution allows the fiber jet to be extensively stretched by mechanical forces without the need of electrical fields. Figure 2d shows an array of 10-cm-long PEDOT:PSS fibers being suspended over a substrate-free plastic frame, and the fiber array could be used to connect and power a light-emitting diode (LED) with a power supply. Figure 2e shows typical PEDOT:PSS fibers in comparison to a human hair, and the insert micrograph shows the fiber has a smooth surface texture. The AgNP fibers have a rough surface texture because they are composed of agglomerated silver nanoparticles (Fig. 2f). The Energy-dispersive X-ray spectroscopy (EDS) mapping in the insert of Fig. 2f further confirms that the fiber is rich in silver content (around 58% in weight).

**Fig. 3:**
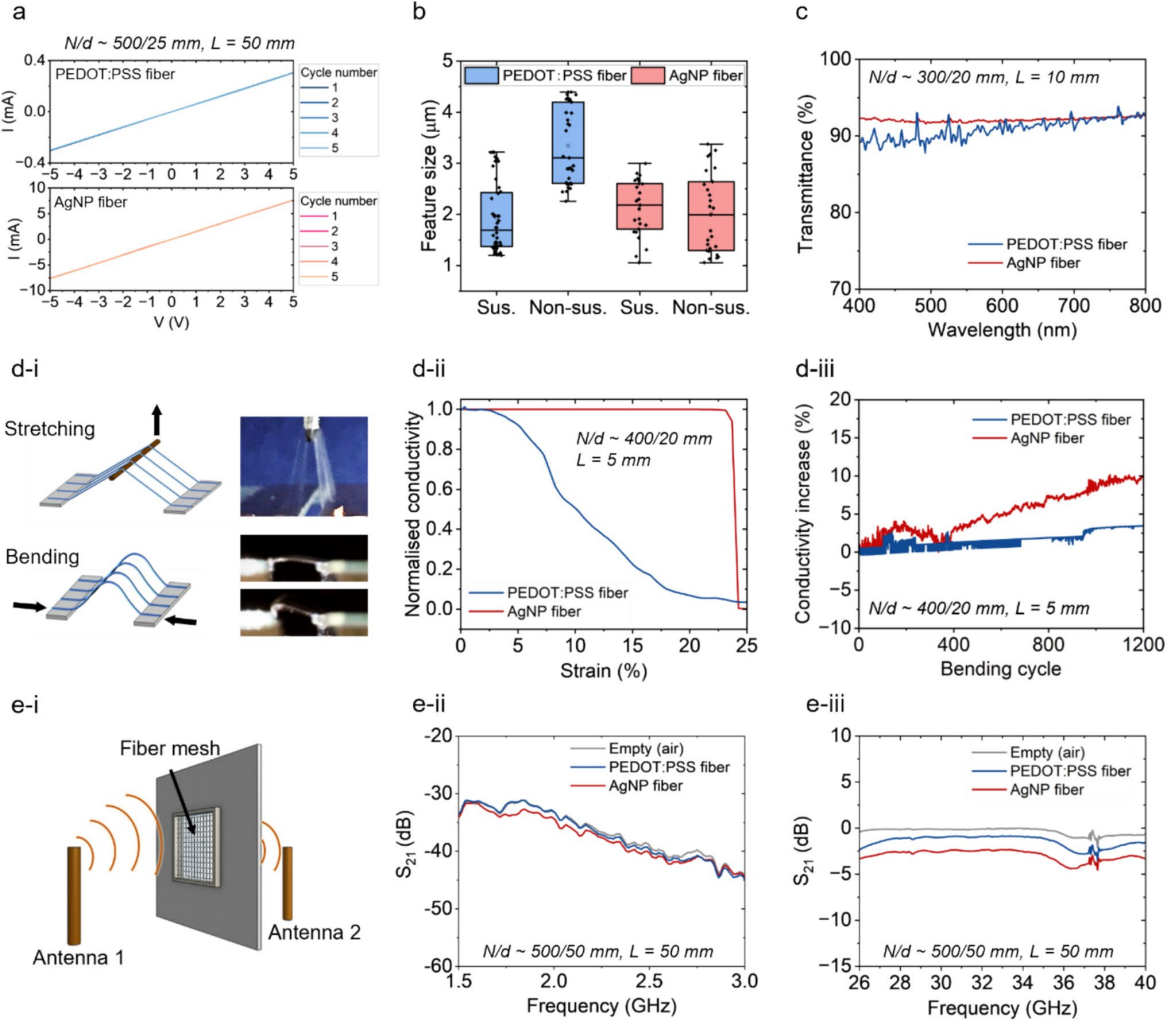
Optical, mechanical, and electromagnetic wave transmittance properties of the microfiber arrays. **a** Cyclic voltammetry measurements on an array of PEDOT:PSS or AgNP fibers (*L* = 50 mm, *N/d* ~ 500/25 mm). **b** Statistical analysis of the fiber feature size (sus., suspending, non-sus, non-suspending, the body of the box represents 25–75% of the dataset, the middle line inside the box represents the middle number, and the error bar represents the rage of the dataset). **c** Transmittance spectrum of an array of fibers (*L* = 10 mm, *N/d* ~ 300/20 mm). **d**-**i** Schematic illustrations showing the mechanical measurement set-ups for characterizing the electromechanical behaviors of the fibers (*L* = 5 mm, *N/d* ~ 400/20 mm). **d-ii** Strain versus conductivity of the fibers during tensile experiments. **d-iii** The change of fiber array conductivity during bending of substrate-less fiber arrays. **e-i** A schematic illustration of measurement set-up for S_21_ properties of the fibers network, composed of two perpendicular fiber arrays in both horizontal and vertical directions (*L* = 50 mm, *N/d* ~ 500/50 mm). **e-ii** S_21_ properties of PEDOT:PSS and AgNP fibers in the low gigahertz range. **e-iii** S_21_ properties of PEDOT:PSS and AgNP fibers in the middle gigahertz range

The high conductivity, microscale size, and EM transparency of the microfibers enable them to be used as unobstructive functionalization layers, which would minimally obstruct the original appearance and functionalities of the base objects. As shown by the cyclic voltammetry measurements in Fig. 3a, both PEDOT:PSS and AgNP fibers exhibit stable and linear ohmic behavior. The stability of microfibers is further tested for a one-week period (Figure S5). The conductivity of the PEDOT:PSS and AgNP fibers could reach ~ 2 × 10^3^ S/m and ~ 1 × 10^5^ S/m respectively, and such conductivity levels are comparable with conducting fibers reported in literature that are made from PEDOT:PSS or silver as their main conducting phases [25, 30, 31]. The average feature size of the microfibers ranges from around 1.5 μm to 3 μm, which also affects its electrical properties such as conductivity (Figure S6). For PEDOT:PSS fibers (Fig. 3b), the feature size of the suspending fibers appears to be much smaller than the non-suspending (i.e., deposited on surfaces) fibers which could be due to the surface wetting phenomenon upon deposition (Figure S7a). The feature size of AgNP fibers appears to have less discrepancy between suspending and non-suspending fibers (Figure S7b). This is because the solvent of AgNP fiber solution is more volatile compared to that of the PEDOT:PSS fibers, making the fibers ‘drier’ upon deposition. Unlike conventional electrospun nanofiber meshes that are usually composed of densely packed fibers, the microfiber patterns created by in situ adaptive fiber deposition are sparsely patterned with large network opening (i.e., the fiber spacing is in the range of tens of micrometers). Therefore, a typical microfiber array is visually transparent with transmittance for visible light at around 90% (Fig. 3c).

The mechanical properties of the microfibers were measured on suspended fiber arrays with home-designed tensile and bending rigs (Fig. 3d-i). As shown in Fig. 3d-ii, the conductivity of PEDOT:PSS fibers would decrease upon stretching, becoming non-conducting at ~ 25% strain; while the conductivity of AgNP fibers remains relatively stable until ~ 20% strain and the conductivity rapidly drops to 0 (Figure S8). Both types of microfibers demonstrate stable performances during repeated bending (Fig. 3d-iii). This is because the small fiber diameter ensures that the tensile stress induced by bending is also very small (Figure S9). Over more than 1000 bending cycles, the conductivities of the fibers remain stable, with slight increase, which could be caused by mechanical sintering effect [32].

The small diameter of the microfibers and the large fiber network openness not only enhance the permissiveness and optical transparency but also minimize the absorbance of EM waves from the lower to middle gigahertz (GHz) range. Conducting microfibers have been found to be highly permissive to electromagnetic (EM) waves in the gigahertz range due to their small size and large network openness [33, 34], so their deposition on electronic devices would minimally obstruct wireless communications. The EM wave transmitting efficiency (*S*_21_) of the fibers was measured with a transmitter antenna and a receiver antenna placed on either side of a fiber network (Fig. 3e-i, Figure S10). A 5 cm × 5 cm fiber network was used, consisting of ~ 1000 fibers for each horizontal and vertical direction. In the lower GHz range, both types of fibers exhibit similar transmittance in comparison to air (Fig. 3e-ii and e-iii). As electronic devices become increasingly prevalent in our daily lives, effective communication among these devices is crucial [35–37]. Conventional electrodes and many wearable devices made with metallic materials could interfere with the EM transmissions [38–41], which might be a disadvantage on some occasions because the EM-opaque devices could block wireless communications. In contrast, the microfiber layers would minimally obstruct wireless communications, promising large-area and smart device-compatible integrations.

## Microfiber Arrays as Permissive Moisture Flow Sensor

**Fig. 4:**
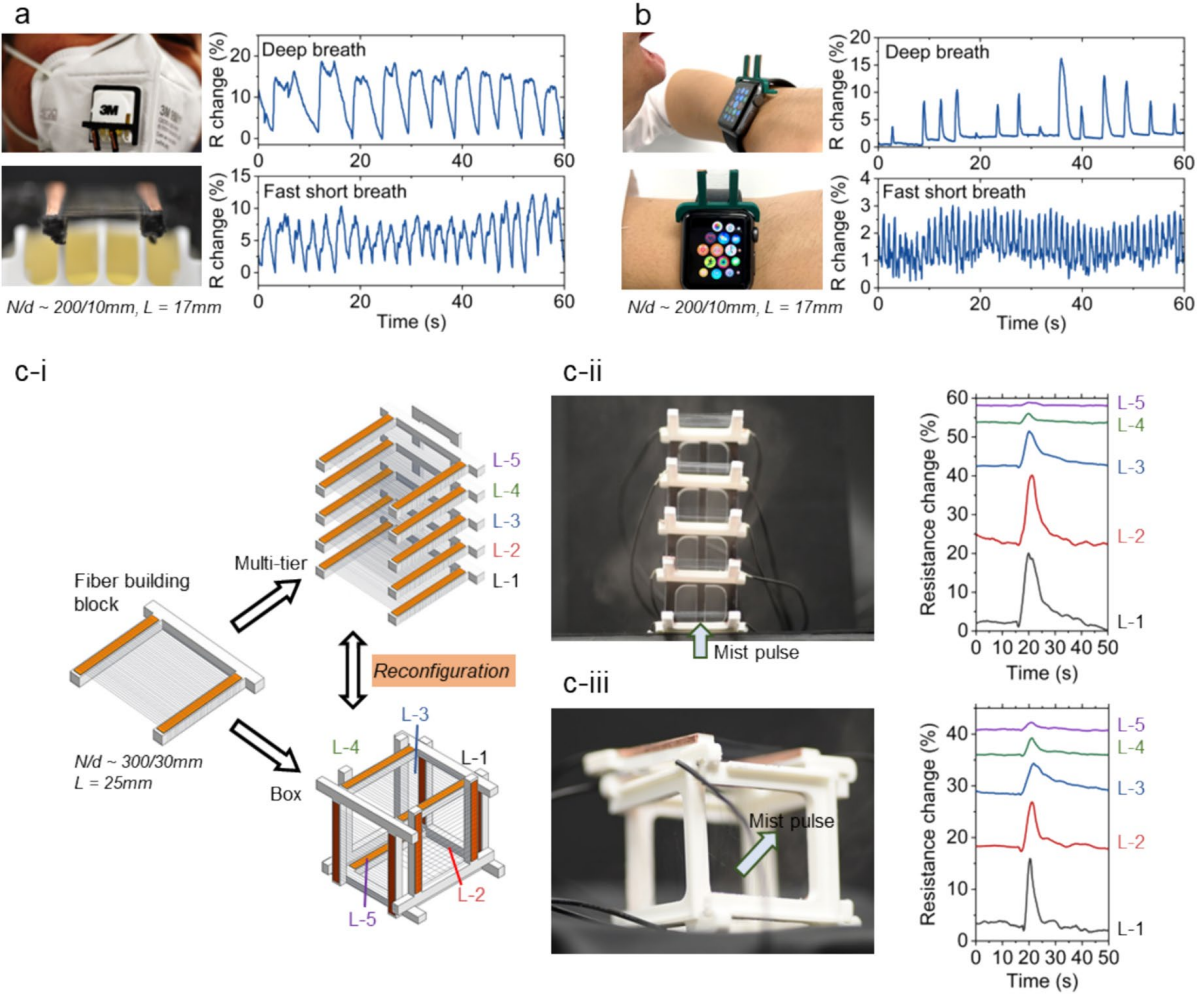
Designable and reconfigurable PEDOT:PSS microfiber patterns for flow sensing. Substrate-less PEDOT:PSS microfibers (*L* = 17 mm, *N/d* ~ 200/10 mm) could be printed onto specially-designed 3D printed frames that could be mounted onto **a** the valve of a face mask and **b** an Apple Watch to enable breath pattern detection. **c** Assembling microfiber building blocks for reconfigurable flow sensors. **c-i** Schematic illustrations showing the microfiber “Lego-like” building blocks to be assembled into a multi-tier design and a box design, and the blocks could be reassembled between the two designs. **c-ii** A multi-tier moisture flow sensor with mist pulse applied from bottom, and the fiber array resistance change in each layer. **c-iii** A box moisture flow sensor with mist pulse applied from bottom to top right, and the fiber array resistance change in each façade of the box (*L* = 25 mm, *N/d* ~ 300/30 mm for each fiber ‘Lego’ block)

Small-diameter fibers have large surface-area-to-volume ratio, enabling rapid moisture absorption and dissipation. Therefore, compared to film-based moisture flow sensors, the substrate-less microfibers could be more responsive to the rapid dynamics of moisture flow [25]. We show that the substrate-less PEDOT:PSS microfibers could be deposited onto various kinds of FFF (fused filament fabrication) printed plastic frames that allow them to be interlocked with everyday objects. As shown in Fig. 4a, a frame with printed PEDOT:PSS fiber arrays could be mounted onto the valve of a face mask, thereby equipping the face mask with the capability to monitor breathing patterns and frequencies, including normal and rapid breathing. The resistance of the PEDOT:PSS microfibers would change in response to humidity variations [25]. In humid conditions, while the resistance increases, the tensile strength of the microfibers is also weakened by the moisture in air (Figure S11). When the face masks are to be changed or disposed, the frames could be easily detached and fibers cleaned for reuse or recycle. Similarly, frames with a different design could fit onto smartwatches as shown in Fig. 4b for breath detection. When the fibers are damaged or wiped off, new fibers could be reprinted onto the recycled plastic frames. In addition, the fibers could also be directly printed onto the screen of the smartwatch, and this could enable water moisture and ammonia vapor detection (Figure S12). In both examples, the fiber arrays are optically transparent, not affecting the displays of the smartwatch screen. The EM wave transparency of the fiber arrays means the Bluetooth and Wi-Fi communications of the smartwatch would not be blocked even with the microfiber electrodes covering its body.

Conventional 3D microfiber structures built with fiber spinning and FFF hybrid printing techniques are usually permanent and inflexible [42, 43] and the FFF-printed plastic frames are usually difficult to reuse if the microfibers are damaged. Here, we further show this “Lego-like” assembly strategy to construct 3D microfiber architecture that facilitates reconfiguration and reuse of the individual microfiber frames. The suspending PEDOT:PSS microfibers arrays were patterned onto FFF-printed plastic frames ($\theta$ ~ 58$^\circ$), which were designed with interlocking features for assembly. The frames could then be used as “building blocks” to construct the 3D moisture flow sensing architectures, as exemplified by a multi-tier and a box design. The fiber “building blocks” could be reconfigured between these two designs. Figure 4c showcases that both designs of the 3D moisture flow sensors could detect spatial moisture flow dynamics in different configurations. The small diameter and permissiveness of the microfiber electrodes ensure that the moisture flow could freely pass through the fiber blocks while being detected. The fiber arrays could be easily wiped off from the FFF printed frames using a wet tissue after use, and the estimated fiber waste generated from each ‘building block’ frame is ~ 0.025 mg (Note S1). The frames could then be reused for further fiber depositions.

Despite that far-field electrospinning and blow spinning could also produce substrate-less micro- or nanofiber meshes [19, 21, 44], these techniques may fall short in creating highly ordered fiber arrays with large network opening due to the jet whipping phenomenon. In a high static field, the charged fiber jet could experience lateral instability and start to whip [45, 46]. This whipping could greatly compromise the capability to control the deposition of microfibers during electrospinning, preventing the achievement of high-precision fiber patterning. In contrast, in the adaptive fiber deposition strategy, the microfibers are initiated and deposited via mechanical shearing and stretching. Compared to the randomly-stacked thick fiber meshes, the uniformly parallel fiber arrays with an average fiber spacing of around 100 μm, as shown in Fig. 4c, could be more permissive to the moisture flows especially when stacked into several layers. As illustrated in Supplementary Table 1, the design parameters of the uniform fiber arrays could be tuned on-demand to adjust their electrical and optical properties. In contrast, these parameters would be less tunable with the non-patterned fiber meshes because the conduction path of each fiber is different. In addition, the uniformly parallel fiber arrays, created by adaptive fiber deposition, could potentially enable high spatial resolution sensing by separating the contact electrodes in the future (Figure S13).

## Functionalization to Enable Daily Biopotential Monitoring

**Fig. 5:**
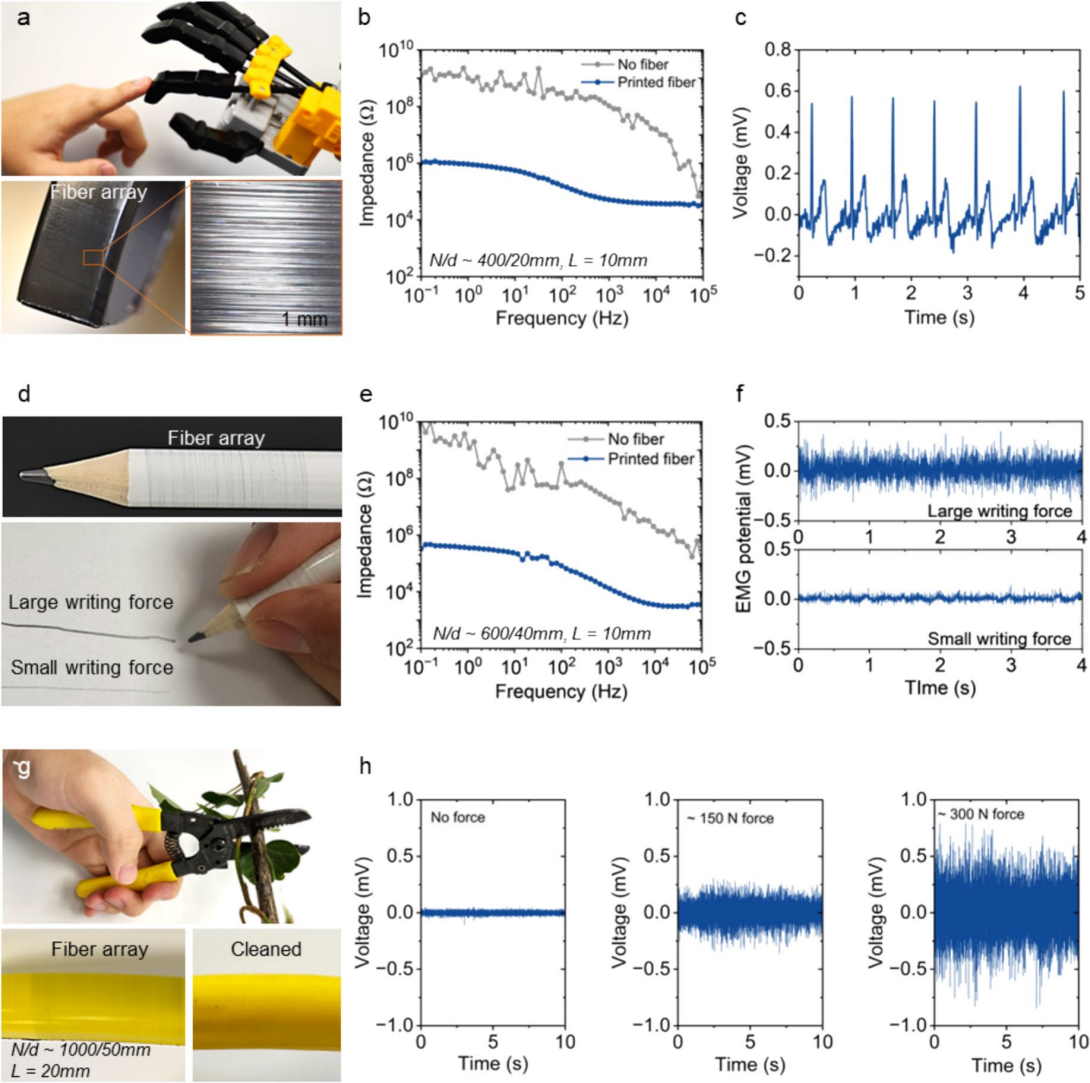
The spin-on PEDOT:PSS microfiber electrodes to enable real-time biopotential measurement from a robotic hand, a pencil, and a plier tool. **a** Pictures showing the index finger of a robotic hand with printed microfibers to be in contact with a human index finger (*L* = 10 mm, *N/d* ~ 400/20 mm). **b** Contact impedance between the robotic and human fingers before and after fiber printing. **c** Typical ECG signals measured from the robotic finger when in contact with human finger. **d** Pictures showing a pencil wrapped by the microfibers to be used for writing under small and large forces (*L* ~ 10 mm, *N/d* ~ 600/40 mm). **e** Contact impedance between the pencil and human fingers before and after fiber printing. **f** In situ EMG recordings from the fingers when writing with the pencil under small and large forces. **g** Pictures showing a plier with printed microfibers onto one of the handles (*L* ~ 20 mm, *N/d* ~ 1000/50 mm). **h** EMG signals when using the pliers with various force levels

The PEDOT:PSS microfiber electrodes could functionalize a range of everyday objects to enable them for biopotential monitoring when in contact with human bodies. Monitoring biopotentials, such as electrocardiogram (ECG) and electromyography (EMG), in a daily basis could be important for health management [47] and early diagnostics [48]. Specialized biopotential measurement tools, such as hydrogel patches, could restrict our normal activities, and their shelf and active lifespan might suffer from dehydration issues. Here, we show that the microfiber electrodes could be adaptively decorated onto the surfaces of a range of existing objects, enabling them to possess suitable skin interfacial impedance for biopotential measurements when held by human. For example, electronic skins with sensing capabilities are crucial to enable the robots and prosthetics to be more human-interactive [7, 49]. However, many robots in service are not equipped with human-interactive sensing capabilities. Without replacing the existing robots, the microfiber electrodes provide a cost-effective approach to rapidly functionalize them with human-interactive sensing functions. PEDOT:PSS microfiber electrodes could temporarily form a conducting dry electronic skin on the robotic finger ($\theta$ ~ 92$^\circ$) (Fig. 5a). Due to the low skin contact impedance of PEDOT:PSS [50], this transient electronic skin could enable the robotic finger to detect biophysiological signals from human when in contact. The contact impedance between the robotic and human fingers would drop significantly after 2–3 min of fiber printing (Fig. 5b), and this could enable ECG measurements (Fig. 5c).

Furthermore, the microfiber electrodes could be attached to a wide range of everyday objects that are in direct contact with human skin. The unobstructive feature enables biopotential monitoring from these objects without impacting their original functions. As shown in Fig. 5d, the microfiber electrode could be printed to wrap the surface of a pencil, enabling EMG detection from the hand muscles when writing. The contact impedance drops after microfiber electrodes are printed on the pencil (Fig. 5e) so that the in-situ EMG signals could be recorded when writing with different force levels (Fig. 5f). In another example, the microfiber electrodes could be transiently wrapped onto the handle of a plier, which is made of insulating rubbers (Fig. 5g). The originally insulating handle could then be functionalized to possess a low contact impedance for ECG measurement when held by hand (Figure S14). The fiber arrays could also enable in situ muscle EMG signal measurements from the thumb muscle area. As shown in Fig. 5h, the EMG signals of different amplitudes could reflect the person using the plier with various force levels.

In the above examples, the unobstructive microfiber electrodes would influence the functions and normal use of the original objects (*i.e.*, pencils for writing and pliers for gardening). Compared to conventional hydrogel biopotential electrodes, which might dehydrate after some time, the dry microfiber electrodes are long-lasting. Once the sensing task is completed, the fibers could be easily wiped-off without damaging or staining the original surfaces of the objects. Additionally, many objects consist of curved surfaces, which greatly challenge the prefabricated planar electronics to conform to surfaces with large curvature [51, 52]. In contrast, the adaptive fiber deposition allows for adaptive fabrication of the electrodes on various objects with large curvatures (*i.e.*, the curvature of the pencil is around 200 m^−1^).

## Microfibers for Energy Conversion and Electronic Connection

**Fig. 6:**
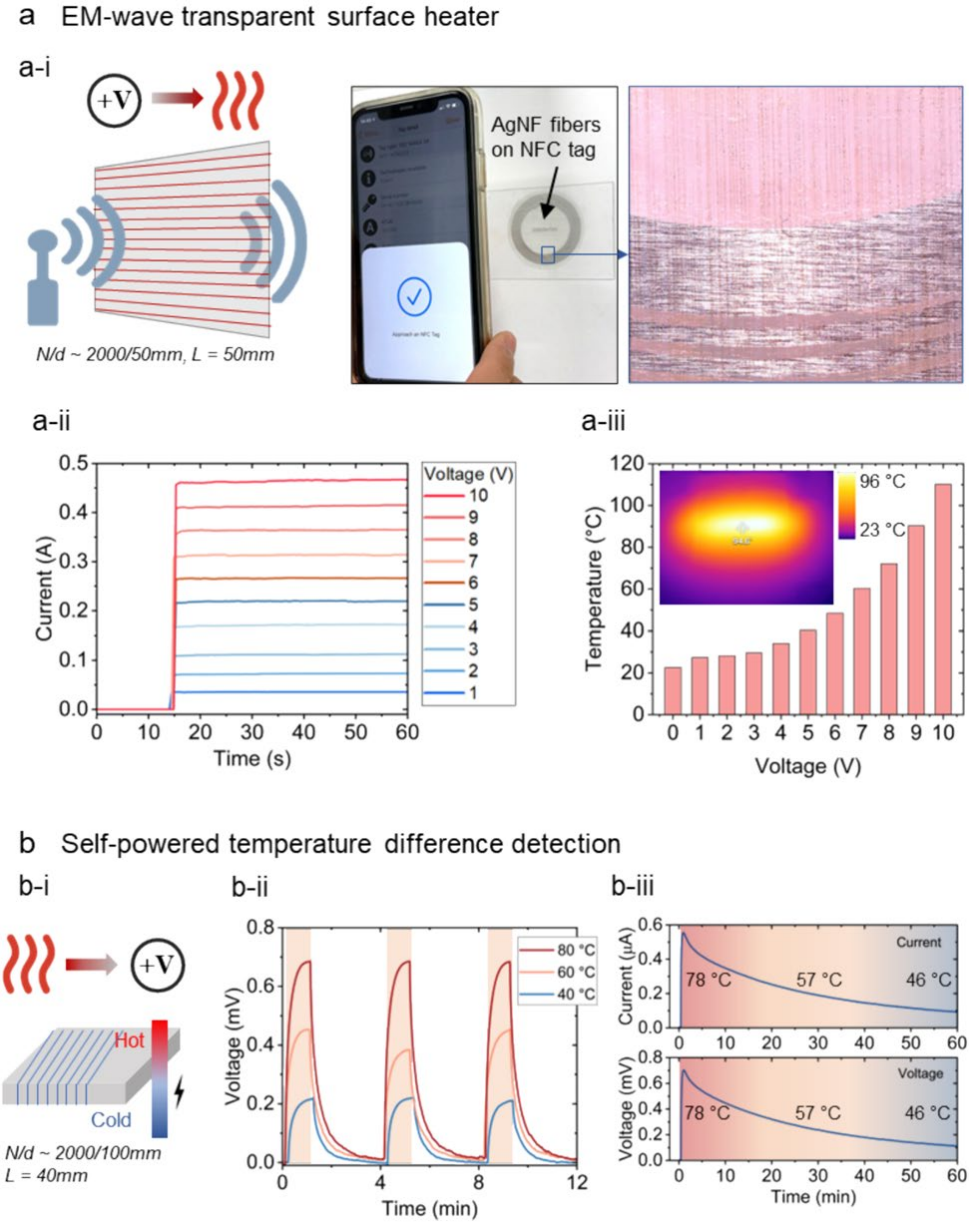
Microfiber electrodes for heating and thermoelectric energy conversion. **a** EM wave transparent surface heaters by AgNP fiber arrays. **a-i** Schematic illustrations of the EM wave transparent surface heater and images showing an NFC tag with printed AgNP fibers (*L* = 50 mm, *N/d* ~ 2000/50). The fibers are visually transparent and do not block the radio-wave transmittance, as evidenced by the detection of the NFC tag from a smartphone, and the fibers could be powered for heating as shown by the voltage-current responses in **a-ii** and heating temperature in **a-iii**. **b** An array of PEDOT:PSS fibers wrapped around a coaster for thermoelectric energy harvesting when a hot container is placed on top of the coaster (*L* ~ 40 mm, *N/d* ~ 2000/100 mm). **b-i** Voltage generated from the coaster when placing a heat mat of various temperatures on the top side of the coaster. **b-ii** Voltage and current generated from the coaster when placing a bowl with hot water of 80 °C and cooling down naturally

The highly conducting and transparent AgNP microfiber array could be used as EM transparent microheaters. Normally, surface heaters require high aerial coverage of conducting materials, which could make the surface opaque to EM waves, potentially blocking wireless communications [53]. For example, the deicing mechanisms on windows could sometimes interfere radio signals [54]. However, the AgNP microfiber array could be used as optical and electromagnetic wave unobstructive microheaters. As shown by an example in Fig. 6a-i, when an array of AgNP fibers were printed to fully cover a near-field communication (NFC) tag ($\theta$ ~ 81$^\circ$), the tag could still be recognized by a smartphone. Fig. 6a-ii shows the heating power characterizations of an array of around 2000 AgNP fibers of 50 mm in length across a distance of 35 mm. The directional resistance of this fiber array is ~ 20 Ω and the fibers could output ~ 4.6 W heating power with 10 V voltage input. The heating efficiency is shown as the voltage temperature characterization in Fig. 6a-iii, where the center of the fibers could reach around 110 °C with 10 V voltage input.

Next, we demonstrate that PEDOT:PSS microfiber array could transform a normal coaster ($\theta$ ~ 73$^\circ$) into one with thermoelectric energy conversion capabilities thanks to the thermoelectric property of PEDOT:PSS [31, 55, 56]. An array of PEDOT:PSS fibers were printed to wrap around a coaster that is made of artificial leather, and when a hot kitchen container was placed on top of the coaster, a temperature difference between the top and bottom surface of the coaster was formed (Fig. 6b and Figure S15). Figure 6b-ii shows the voltage generated from the coaster when a hot plate of controlled temperatures was placed onto the top surface of the coaster. As a demonstration, a glass container with hot water of around 80 °C was placed onto the coaster, and the voltage and current generated from the coaster were recorded for an hour (Fig. 6b-iii). In future works, incorporating state-of-the-art flexible thermoelectric materials [57] into the fibers could potentially enable convenient energy harvesting from various heat sources in our homes and workplaces.

**Fig. 7:**
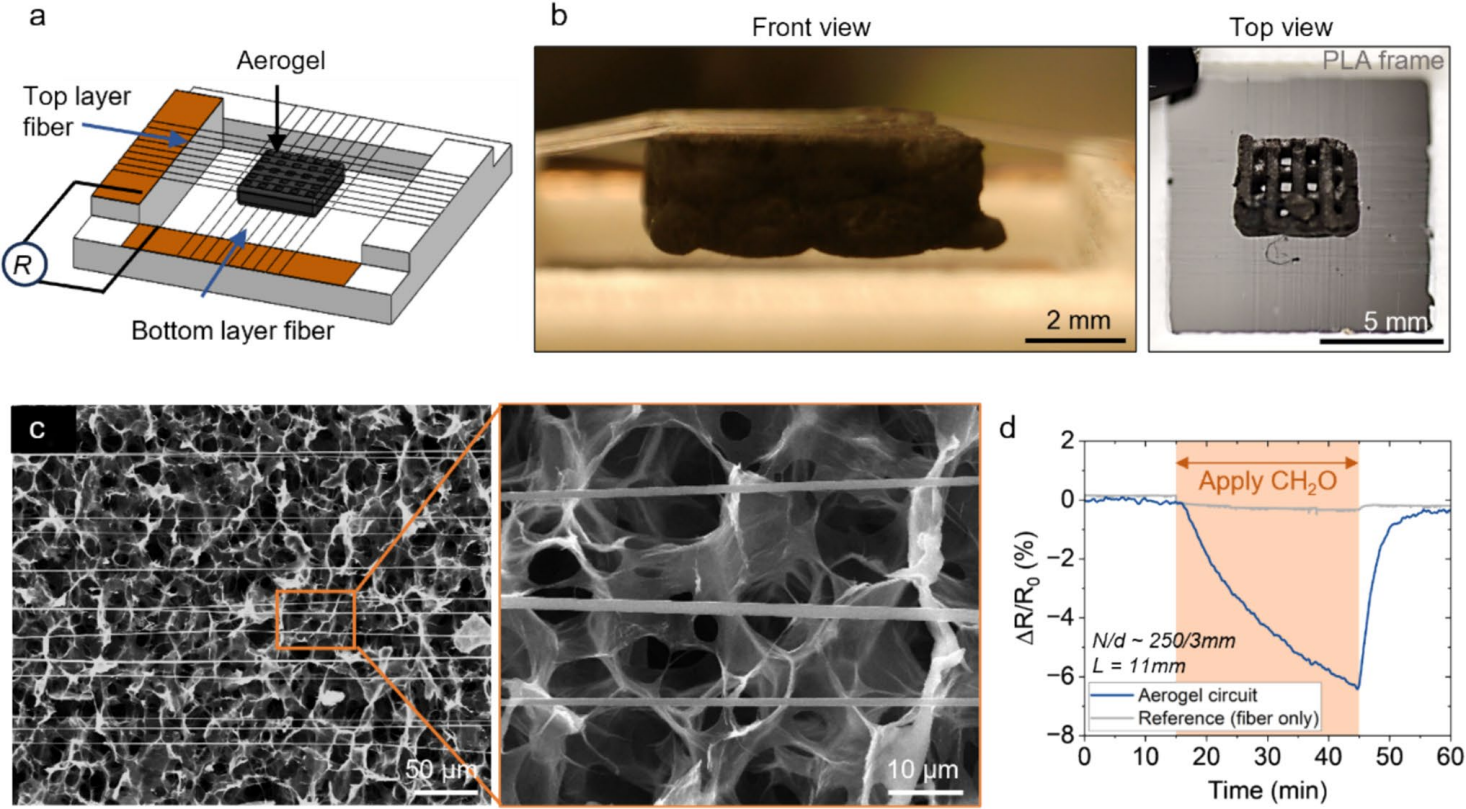
PEDOT:PSS microfiber electrodes to connect graphene-based aerogels for toxic gas sensing. **a** A schematic illustration showing two layers of microfiber arrays “sandwiching” a piece of graphene-based aerogel (*L* = 11 mm, *N/d* ~ 250/3 mm for both top and bottom fiber array). **b** Photos showing the aerogel being connected by microfiber arrays. **c** SEM images showing the microfibers deposited on the surface of the aerogels. **d** The resistance change of the microfiber-aerogel system when exposed to formaldehyde gas

Finally, we show that the microfiber electrodes could be interfaced with unconventional materials that require high permissiveness. Graphene-based aerogels are known as one of the lightest solids in the world attributed to their high porosity [58, 59], which could be used for efficient toxic gas sensing [60]. To connect the aerogels into a circuit, conventional planar and non-permissive electrodes are often used but they would cover the porosity and greatly reduce the gas absorbing and sensing efficiency. The uniformity and success rate of coating or directing painting functional inks onto objects are highly dependent on the surface wettability between the inks and the surfaces [61]. The adhesion between the microfibers and the graphene flakes ensures the functional and structural integrity of the device for transient functionalization (Figure S16). As a demonstration, two layers of PEDOT:PSS microfibers are used to “sandwich” a piece of graphene-based aerogel (Fig. 7a, b). The substrate-less microfiber arrays do not cover the structural porosity of the aerogels (Fig. 7c). The “sandwiched” and suspended graphene aerogels could be used to detect toxic gases such as formaldehyde (Fig. 7d), which is responsive in the resistance decrease, providing an alternative to overcome the obstruction of traditional electrode architecture [26]. In the future, the permissive microfiber electrodes, combined with 3D printed frames, could potentially enable multi-layer graphene aerogel stacking for integrated environmental sensing.

## Conclusion and Outlook

Overall, we demonstrate various applications of using the customizable and conducting microfiber patterns to temporarily enhance the functionalization of a wide range of objects. From daily-use stationaries to smartwatches and unconventional material formats such as porous aerogels, the microfibers are not selective with the geometry or surface textures of the targeted base objects for functionalization. The adaptive printing technique allows for on-demand fiber pattern designability to suit the intended applications.

While providing add-on sensing and electronic connections functions, the microfiber functionalization layers would appear ‘imperceptible’ on the original target base objects. The substrate-less and open-network features ensure that the microfiber patterns would minimally obstruct the original functions and appearance of the target objects. The EM transparency feature of the conducting microfiber patterns makes them suitable for large-area functionalization without shielding wireless communications. In addition, the minimized material and energy consumptions of adaptive fiber deposition ensure that the creation of the microfiber patterns could be scaled up in the future while satisfying cost-effectiveness and sustainability. After service, the microfiber layers could be easily removed without perturbing the original appearance and functionality of the base object. The transient feature of the conducting microfibers helps to minimize the recycling difficulties and enable convenient functional adaptations. Looking ahead, expanding the material library of the microfibers could unlock new applications and enable multi-functional integration, opening a sustainable and customizable “Fiber-of-Things” (FoT) future.

## Supplementary Information

Below is the link to the electronic supplementary material.

Supplementary file1 (PDF 1890 KB)

## Data Availability

The raw data is available upon request with the corresponding authors.
