# Supplementary material for: Adaptive Printing of Conductive Microfibers for Seamless Functional Enhancement Across Diverse Surfaces and Shapes
Source: Adv Fiber Mater. 2025 May 15;7(4):1274–89. doi: 10.1007/s42765-025-00561-6 (PMC12287195; doi:10.1007/s42765-025-00561-6)
Supplement: Supplementary file 1 — Supplementary file1 (PDF 1890 KB) [file 42765_2025_561_MOESM1_ESM.pdf]

Supplementary information for:

Adaptive Printing of Conductive Microfibers for Seamless Functional Enhancement Across Diverse Surfaces and Shapes

Stanley Gong Sheng Ka[a,b,#], Wenyu Wang[a,b,d,#,], Henry Giddens[e], Zhuo Chen[c], Ahsan Noor Khan[e], Yuan Shui[a,b], Andre Sarker Andy[e], Shuyu Lyu[a,b], Tawfique Hasan[c], Yang Hao[e], Yan Yan Shery Huang[a,b,]**

[a] Department of Engineering, University of Cambridge, Trumpington Street, CB2 1PZ, Cambridge, UK

[b] The Nanoscience Centre, University of Cambridge, 11 JJ Thomson Avenue, CB3 0FF, Cambridge, UK

[c] Cambridge Graphene Centre, University of Cambridge, 9 JJ Thomson Ave., Cambridge CB3 0FA, UK.

[d] Thrust of Smart Manufacturing, Hong Kong University of Science and Technology (Guangzhou), Guangzhou, China

[e] School of Electronic Engineering and Computer Science, Queen Mary University of London, 10 Godward Square, E1 4FZ, London, UK

[#] These authors contributed equally to the work.

E-mail: wenyuwang@hkust-gz.edu.cn (Wenyu Wang); yysh2@cam.ac.uk (Yan Yan Shery Huang)

Included in this file:

Note S1

Table S1-S2

Figure S1-S16

Supplementary Note 1. Estimation of energy and materials consumption for microfiber fabrication

The cost effectiveness of the reported microfiber printing approach could be analyzed from the following aspects: first, the total electricity consumed by the setup, such as laptop, Arduino Uno, linear stage, and microfluidics pump, is estimated maximum 20 Wh in 5 minutes for making a fiber device (~200-300 fibers), which would cost less than GBP 0.10. The mass of a microfiber device is ~ 0.01 to 0.4 mg, considering the fiber diameter is only a few μm. The material used to produce a microfiber array is then only a few μL from the fiber solution, in which the estimated cost for 1kg fiber solution is as follows: GBP 600 for PEDOT:PSS fiber solution (PEDOT:PSS sold ~ GBP 400 and PEO ~ GBP 200 commercially) and GBP 200 for AgNP fiber solution (silver acetate sold ~ GBP 130, ammonium hydroxide ~ GBP 40, and formic acid ~ GBP 30). Thus, adding up other miscellaneous costs such as logistics and labor (national minimum wage ~ GBP 12 per hour), the estimated cost is GBP 2.00 per device.

Table S1. Key design parameters of the conducting microfiber patterns used in various applications. This table includes the fiber number density across the width (*N/d*), the fiber length (*L*), and the rationale for choosing the *N/d* and *L*.

**Applications**	**Figure**	***N/d* (mm)**	***L* (mm)**	**Estimated transmittance (%)***	**Estimated resistance *R* (kΩ)****	**Rationale for choosing the *N/d* and *L***
Flow sensing using face mask and Apple watch	4 (a) and (b)	200/10	17	91 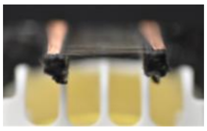 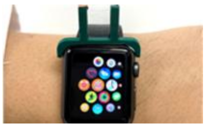	12	From Fig. 4 (a), ~ 200 fibres could cover the entire valve of the face mask. The length (17mm) was chosen to fit to the plastic frame used.
Flow sensing in building blocks	4 (c-i)	300/30	25	94 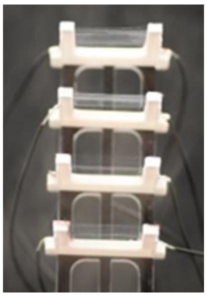	12	~ 300 fibres printed on the block (width of 30 mm and length of 25 mm) are sufficient to sense the mist flow.
ECG via contact of robotic and human fingers	5 (a)	400/20	10	91 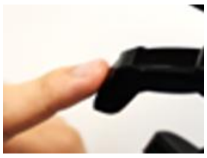	\	Large fibre number and deposition width were chosen to ensure the finger is fully in contact with the fibre array. The length is 10mm because the fiber array is wrapped around the robotic finger.
EMG sensing from holding: pencil and plier	5 (d)	600/40	10	92 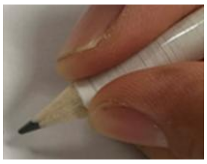	\	Large fibre number was chosen to ensure the maximum contact of skin to the fibres. The lengths of the fiber array are 10mm and 20mm because the fiber array are wrapped around the pencil and plier.
	5 (g)	1000/50	20	91 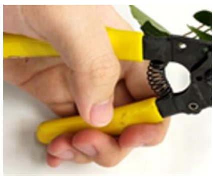	\	

Electromagnetic wave transmittance by AgNP fiber arrays	6 (a)	2000/50	50	90 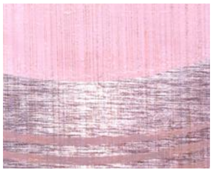	0.08	~ 2000 fibres are sufficient to cover the entire NFC tag. The length is 50mm because the NFC tag is 50mm long.
Thermoelectric energy harvesting	6 (b)	2000/10	40	89 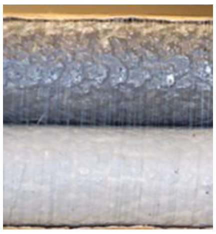	3	~ 2000 fibres are sufficient to cover the surface of the coaster. The length is 40mm as a result of the thickness of the coaster.
Formaldehyde sensing with fibers-sandwiched graphene-based aerogel	7 (a)	250/3	11	90 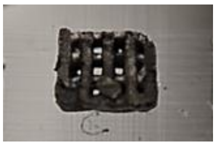	6	A narrow deposition width was chosen to ensure the contact between fibres and the aerogel. The length of fiber array is 11mm long to hold the aerogel (~ 5mm x 5mm) in air.

*based on relationship of transparency and fiber number density published in previous paper[1]. he transmittance, T (%) could be estimated by the equation, T = $0.045 \frac{d}{N} + 89$ where the transmittance limit is 89% when $\frac{N}{d}$ is large.

**Fiber array resistance was estimated in the application cases where the fibers were used as a resistive-based sensor or for electronic connections. The estimated fiber array resistance R is calculated based on the equation $R = \frac{L}{\sigma AN}$, where L is the fiber array length, σ is the fiber conductivity (the conductivity of the PEDOT:PSS and AgNP fibers are taken as 2×10^3 S/m and 1×10^5 S/m respectively), A is the cross-sectional area of fiber, and N is the fiber number. The A is estimated by assuming the fibers have a round cross-sectional area and the diameter is around 2μm.

Table S2. Comparison of other literature to this work. The fabrication process, fiber materials, electrical properties, size, functionality, and applicable surfaces are compared to showcase the versatility of the on-demand printed microfiber.

Reference	Fabrication process (in-situ or ex-situ)	Fiber type and conductivity	Fiber diameter	Function embedded or coated	Applicable surfaces
Composites Part A: Applied Science and Manufacturing **167**, 107427 (2023)	Ex-situ:<br>Transfer required after fabrication	SWCNTs/epoxy-coated E-glass fiber<br><br>Conductivity not reported	~ 21.5 μm	Function coated	/
Fibers and Polymers **19**, 1064-1073 (2018)	Ex-situ:<br>Transfer required after fabrication	Polyvinyl alcohol (PVA)/ PEO/carbon black coated cotton<br><br>σ ~ 80 S/m^2	/	Function coated	/
ChemistrySelect **4 (40)**, 11748-11754 (2019)	Ex-situ:<br>Transfer required after fabrication	Silver brush-coated cotton<br><br>σ ~ 1250 S/m^2	/	Function coated	/
Science **377**, 180-185 (2022)	Ex-situ:<br>Transfer required after printing on collection mandrel	Polycaprolactone (PCL)<br><br>Conductivity not reported	~ 0.9 μm	/	Plastic
Nature Electronics **7**, 586-597 (2024)	In-situ: printed on target surface	PEDOT:PSS<br><br>σ ~ 5000 S/m	~ 2 μm	Function embedded	Human skin, chicken embryo, and leaf

This work	*In-situ: printed on target surface*	*PEDOT:PSS and Ag*<br><br>$\sigma_{PEDOT:PSS}$ *~ 2000 S/m*<br>σ_{Ag} *~ 1x10*^{5} *S/m*	*~ 2* μm	*Function embedded*	*Glass (Fig.6a), plastic (Fig. 4 & Fig.5), leather (Fig. S11), aerogel (Fig. 7)*

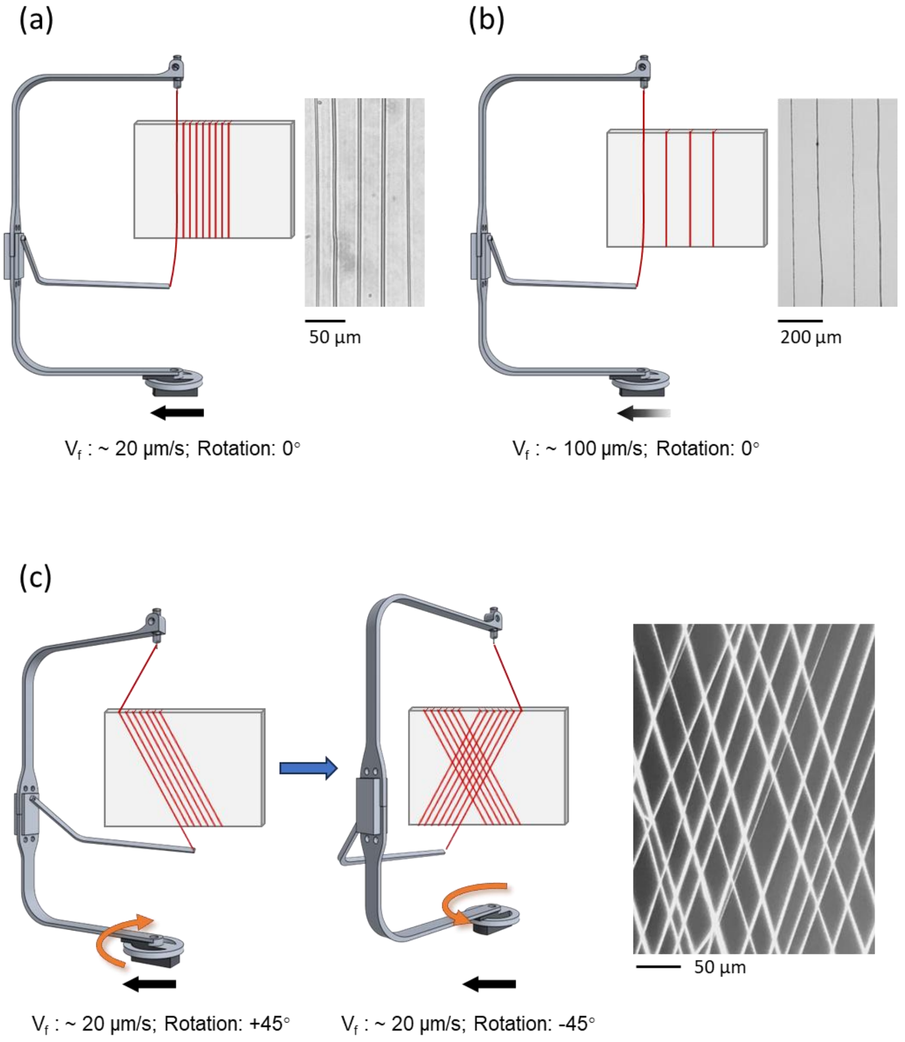

Figure S1. Designing the fiber pattern during freeform fiber deposition. By programming the movements of the base stages, fiber patterns of varying densities and directions could be created.

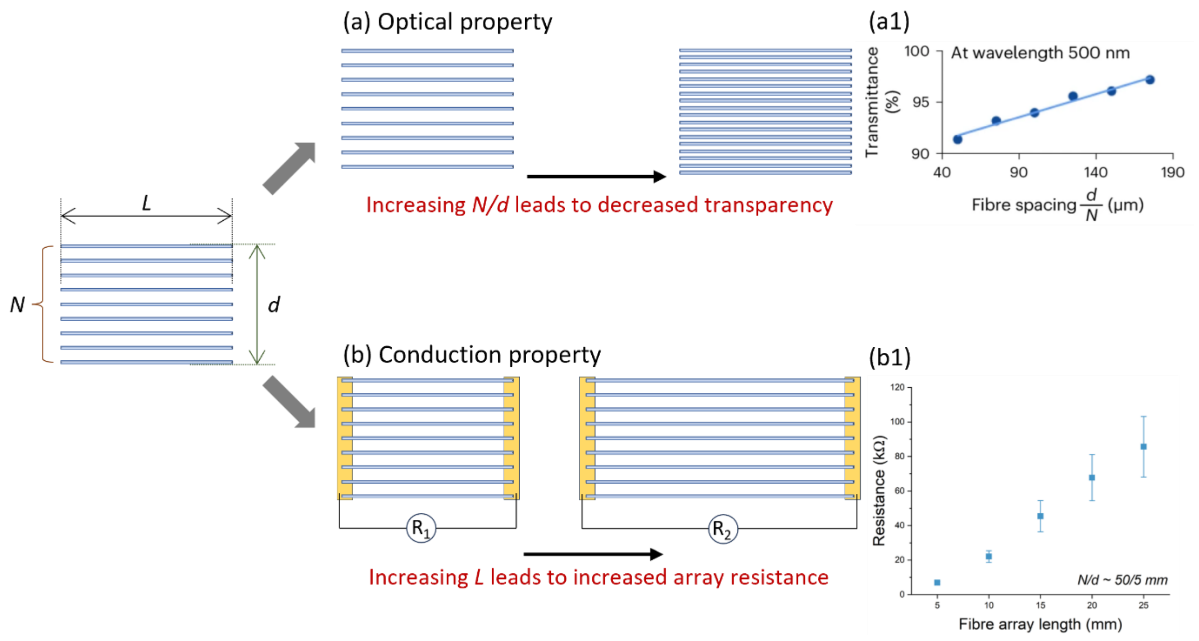

Figure S2. The effect of fiber array design parameters on the optical and electrical properties. The array length (L), array width (d), and the number of fibers (N) are three major design parameters, and their combinations could directly affect the transparency (a) and the resistance (b) of the fiber array. (a1) The relationship of d/N on the transparency of PEDOT:PSS fiber arrays (this figure is adapted from previous findings[1]). (b1) The relationship of fiber array length (L) and the fiber array resistance of PEDOT:PSS parallel fiber arrays (N = 50, d = 5 mm).

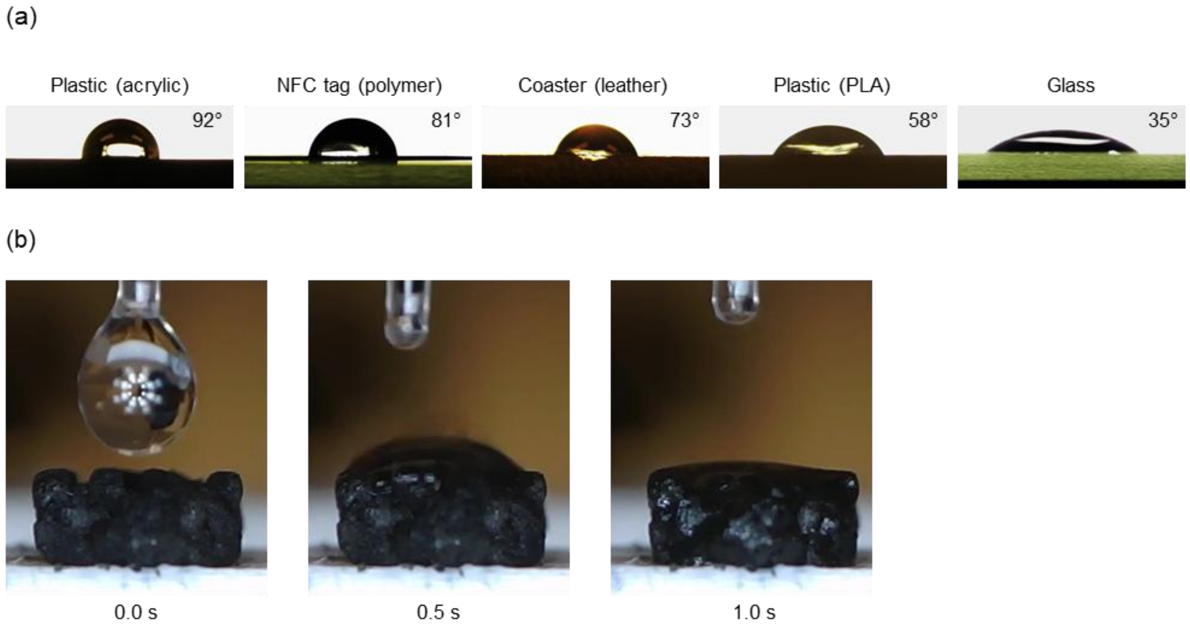

Figure S3. Wetting properties of various surfaces used for coupling with microfiber electrodes. (a) Measurement of water contact angles (θ) on different surfaces of the objects used to serve as substrates for fiber deposition. (b) A photo series showing a droplet of water penetrates into the highly porous graphene-based aerogel within one second of deposition.

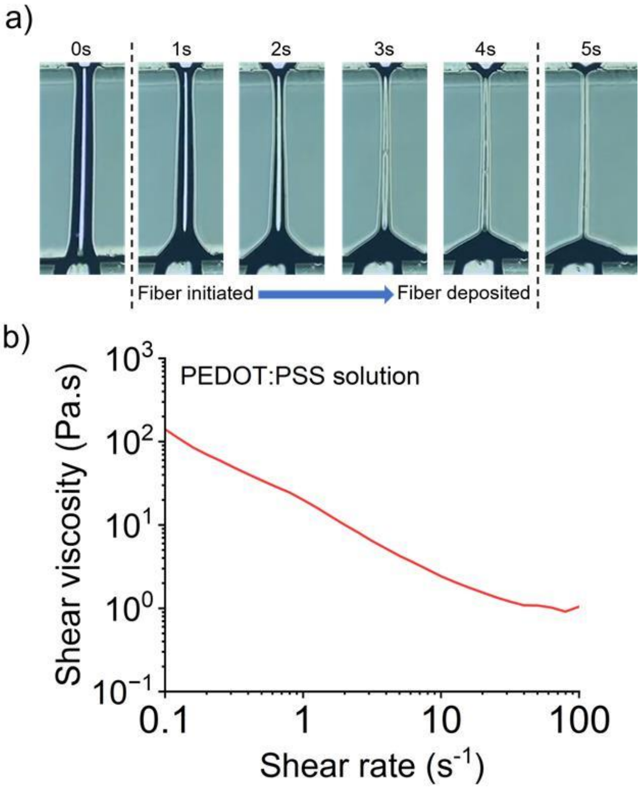

Figure S4. The drying process of a fiber jet upon initiation and its rheological analysis. (a) A sequence of photos showing the fiber jet drying process of a PEDOT:PSS fiber jet, from the moment of being initated by stretcing froce. As seen from the photos, the fiber jet continues to thin upon initiaon, as a result of solvent evaporation. The evaporation would take at least ~ 5 seconds to complete. However, the fiber jet would been deposited onto the surfaces within around 1-2 seconds of initiation. Thus, upon surface deposition, the fiber jet would still remain a semi-wet status. (b) The shear viscosity of PEDOT:PSS fiber solution at shear rate from $0.1s^{-1}$ to $100s^{-1}$. The shear viscosity of the fiber solution reduces as the shear rate increases, indicating the polymeric behavior of the robust long-chain PEO.

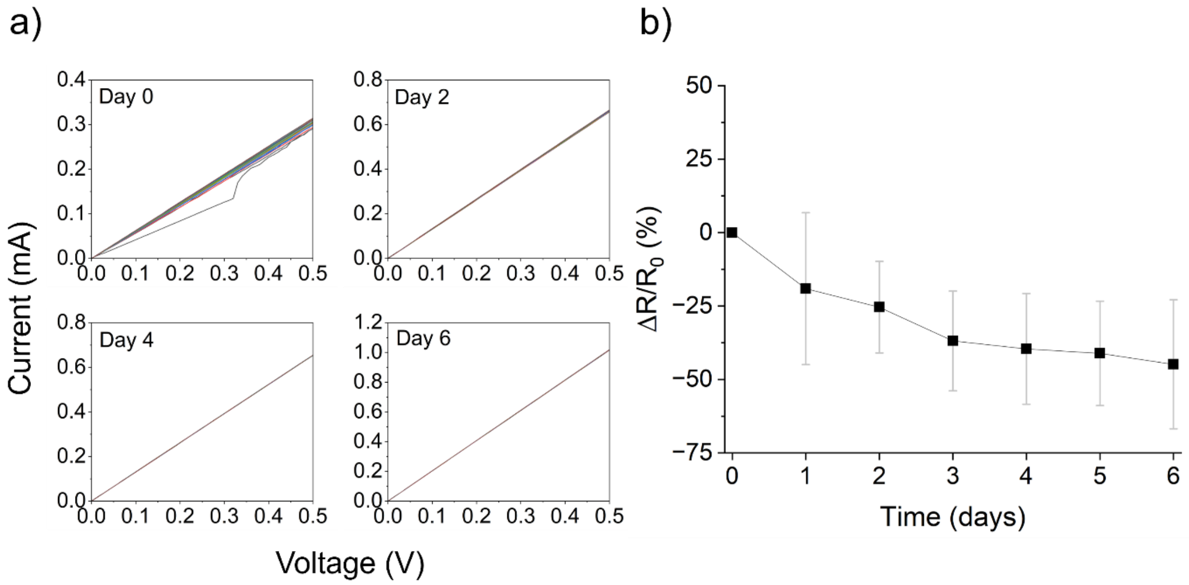

Figure S5. Stability of an AgNP microfiber array in a week. (a) Cyclic voltammetry showing stable ohmic behaviour of an AgNP microfiber array under 0.5V applied voltage for 20 scans (scan rate at 0.2 V/s) from Day 0 to Day 6. (b) Percentage resistance changes over a 1-week period.

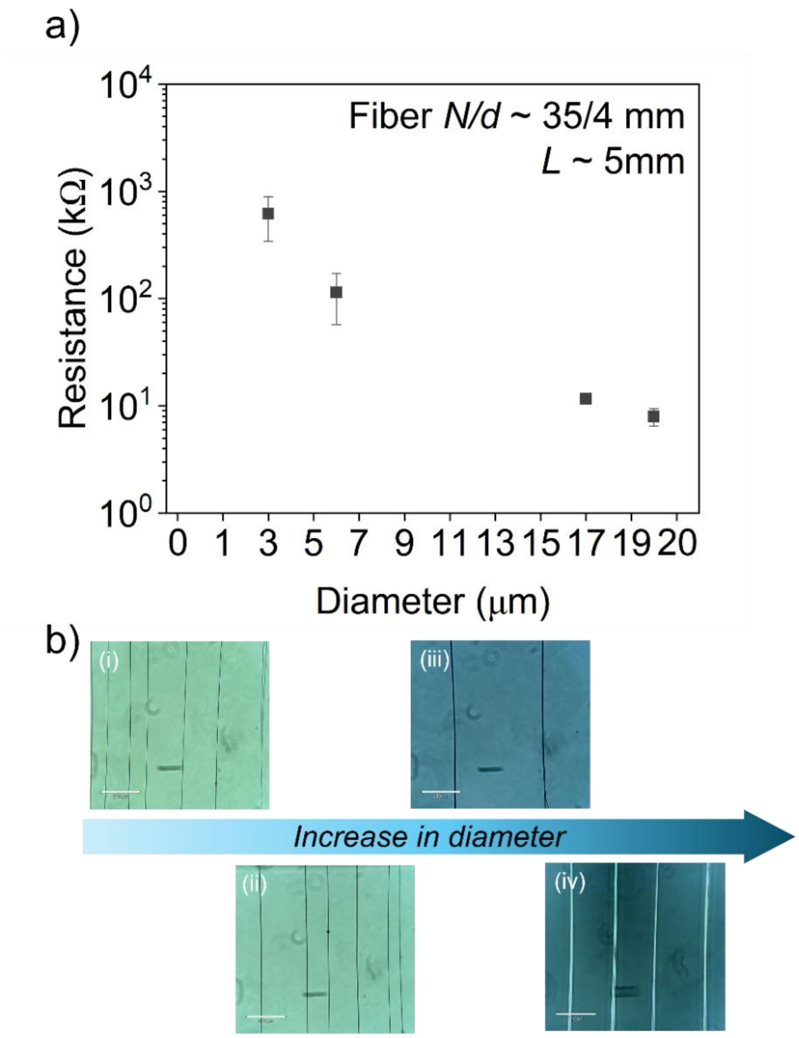

Figure S6. Resistance of microfibers with varying diameters. (a) The average resistance of microfibers (N/d ~ 35/4 mm, L ~ 5mm) showing the resistance drops from 10^3 kΩ to 10 kΩ range while the diameter increases from 1 µms to 10 µms. The electrical behavior of microfibers obeys the resistance equation, $R_f = \frac{\rho L}{AN}$ (R_f is the resistance of a single fiber, ρ is fiber conductivity, L is the fiber length, A is the fiber cross-sectional area (assuming suspending fiber is cylinder), and N is the number of fiber in the array) where it fits the relationship where $R \propto \frac{1}{d^2}$ or $\rho \propto d^2$, and the estimated conductivity should increase with diameter. (b) The microscopic images of microfibers in different diameters: (i) ~ 3µm, (ii) ~ 6µm, (iii) ~17µm, and (iv) ~20µm. The diameter is measured approximately using ImageJ.

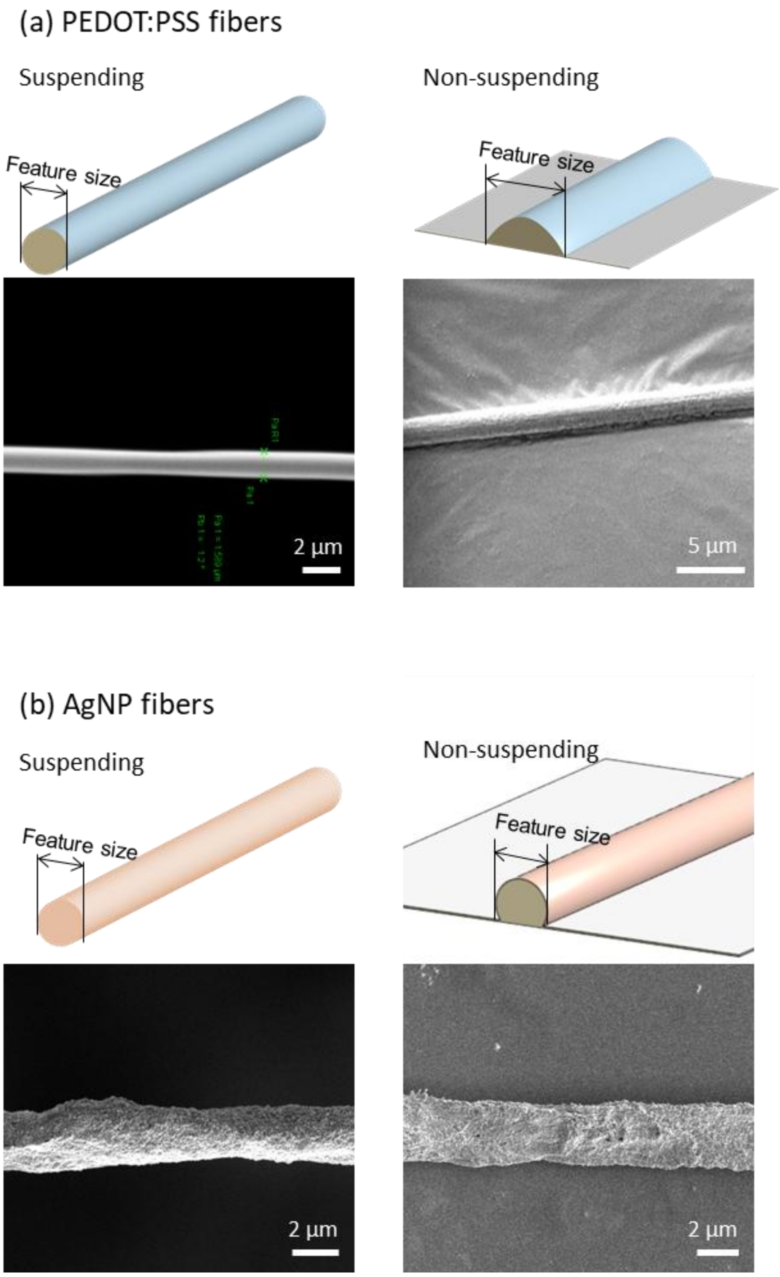

Figure S7. Typical SEM images of suspending and non-suspending (on carbon tape) fibers produced by in situ orbital spinning of (a) PEDOT:PSS fibers and (b) AgNP fibers.

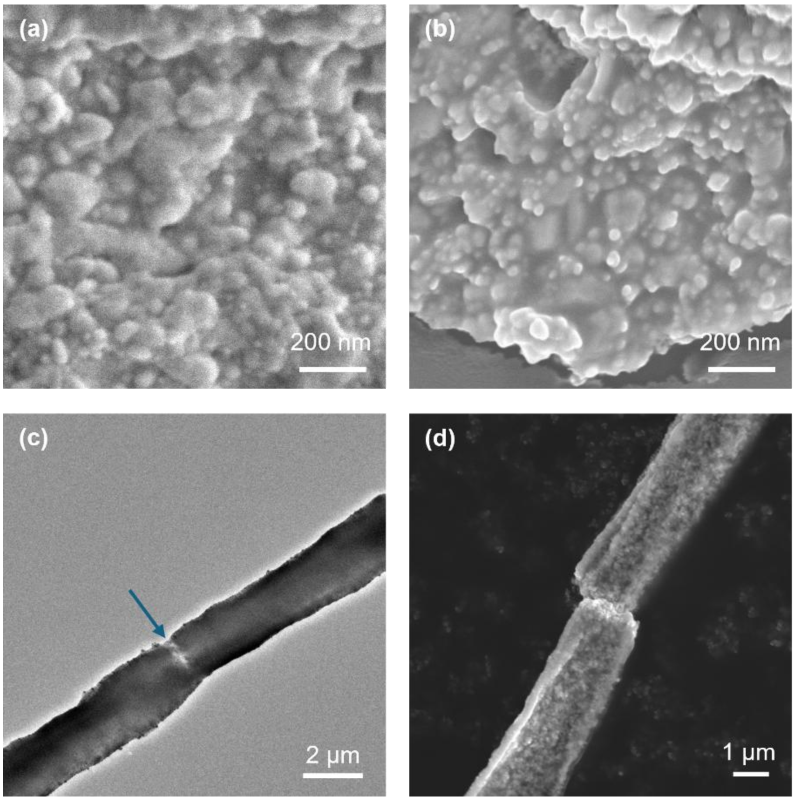

Figure S8. Microscopic images of AgNP microfiber. (a) and (b) Composition of the connected silver nanoparticles in the microfiber. (c) and (d) The fracture of AgNP microfiber causes the disconnection of silver nanoparticles.

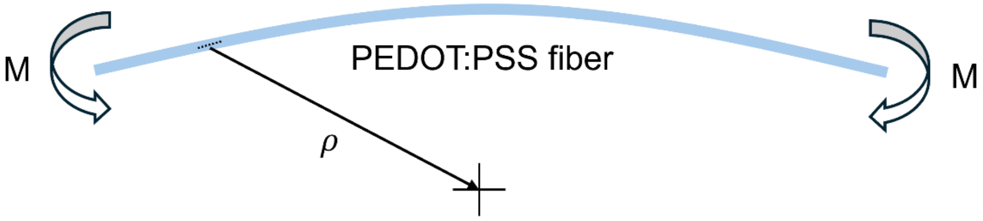

Figure S9. Schematic illustration showing the simplified model to estimate the maximum tensile stress of the suspended fibers during bending. In this model, the individual suspended fiber is simplified as a beam with both ends fixed; thus, the maxima tensile stress (σ) would appear on the top surface of the beam, as estimated from Euler–Bernoulli beam theory, $\sigma = \frac{Ey}{\rho}$, where ρ is the local radius and E is the Young's Modules of the fiber (~ 1 MPa) and y is the distance between the neutral plane and the surface of the fiber (~ 1 μm).

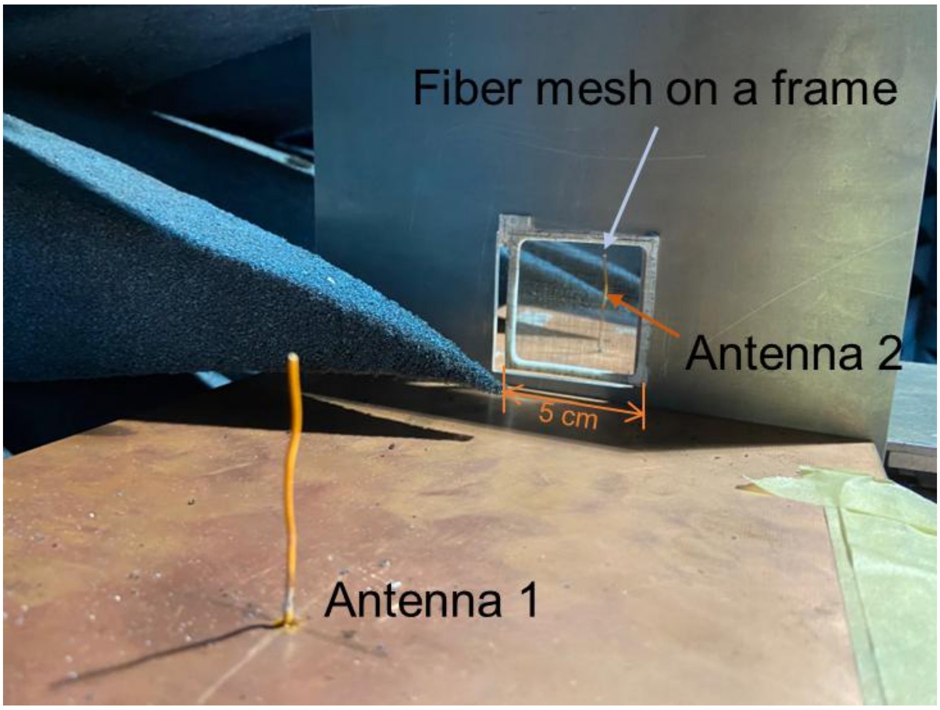

Figure S10. A photo showing the experimental set-up used to measure the transmittance efficiency of electromagnetic waves.

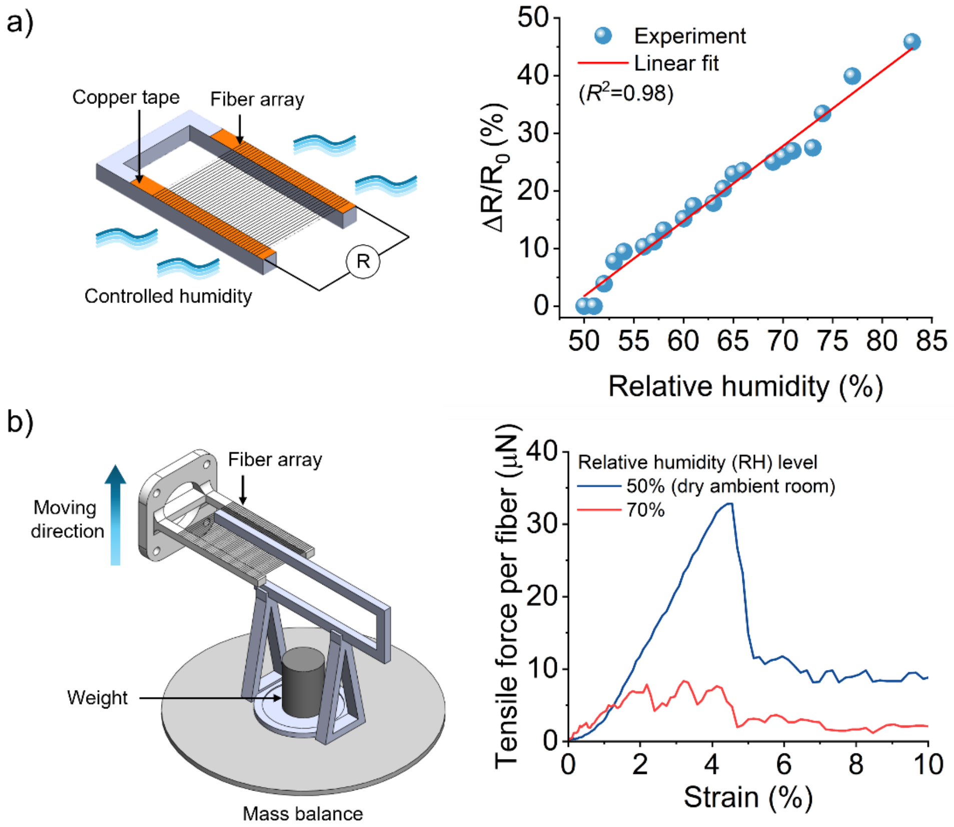

Figure S11. Electromechanical characterization of PEDOT:PSS microfibers in humid conditions. (a) From ambient room RH ~ 50%, the PEDOT:PSS microfiber array (N/d ~ 100/5mm, L~5mm) resistance change is measured with increasing moisture from a humidifier (Aribio). (b) The tensile properties of microfiber array (N/d~200/10mm, L~10mm) in different RH levels. The microfiber array is printed onto a part mounted to a vertical linear stage (Thorlabs MTS50-Z8) When the microfiber array is moving up at 0.05mm/s, the microfibers are pulled by the static arm, which is placed on a portable mass balance (Scout SKX 123, Ohaus) and thus, experiencing tensile force. The strain is estimated by $\varepsilon = \frac{2\sqrt{D^2+\left(\frac{L}{2}\right)^2}-L}{L}$, where D is the vertical displacement of linear stage, and L is the original length of the fiber array.

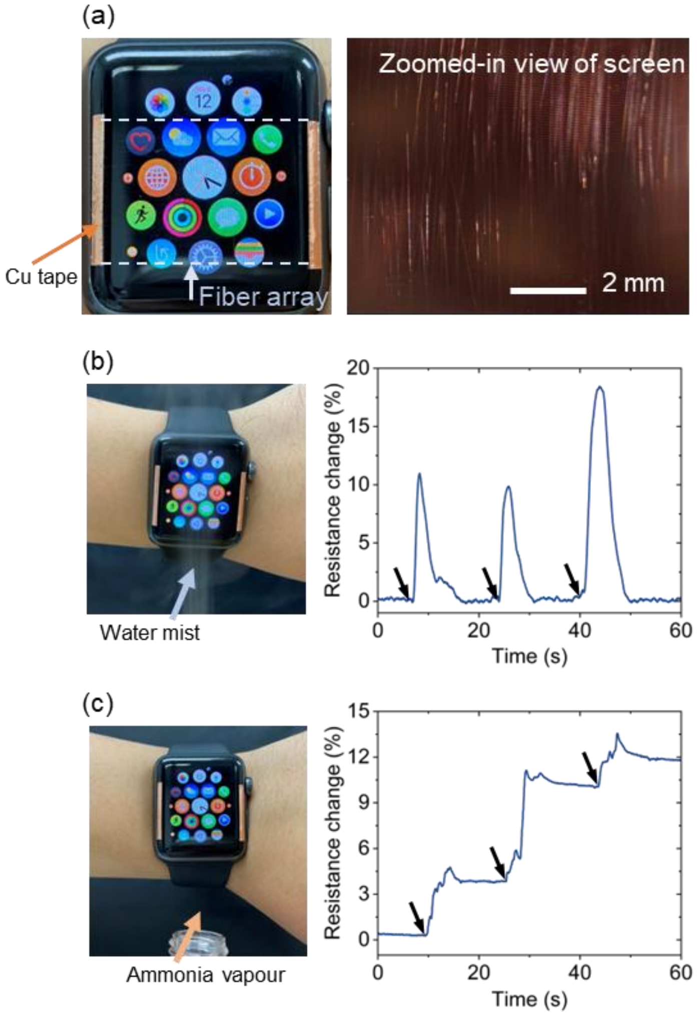

Figure S12. PEDOT:PSS fibers being directly printed onto the screen of the Apple Watch as transparent and transient functionalization for flow sensing. (a) Images showing the printed fibers on the screen of the smart watch. (b) water vapor and (c) ammonia detection with the fiber array (~ 300 fibers of 40 mm length across a distance of 15 mm).

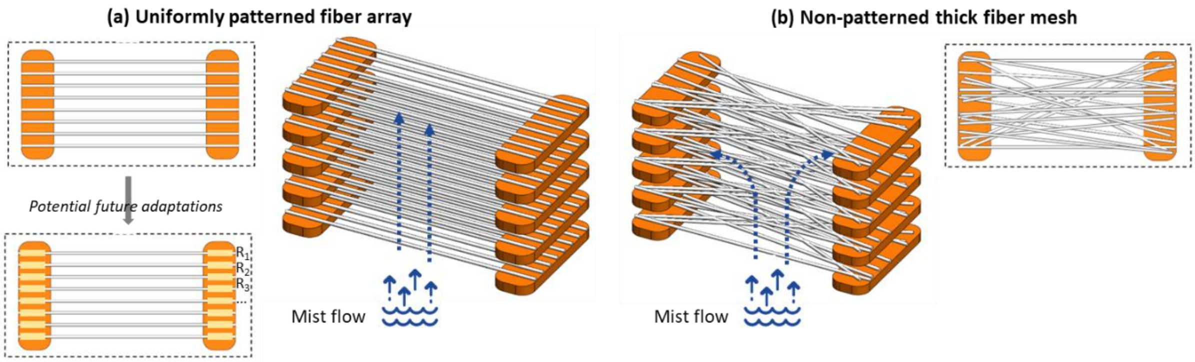

Figure S13. Comparison of uniformly printed fiber array (a) and thick non-patterned fiber mesh (b) in sensing the mist flow. Uniformly patterned microfiber meshes with large fiber spacing (*i.e.*, around 100 μm in Figure 4c) could allow mist flows passing through even if stacked into several layers. In comparison, thick and non-patterned fiber meshes could be less permeable to the mist flow.

Besides, as shown in the Supplementary Table 1, the design parameters, *N/d* and *L*, are customizable with the uniform fiber array depending on the electrical, optical, and mechanical requirements. Potentially in the future, the connection pads of the parallel fiber arrays could be individually separated for high spatial resolution sensing. However, with fibers in non-patterned mesh structures, the electrical path of each fiber is different between the electrodes and the parameters are less tailorable in mesh format.

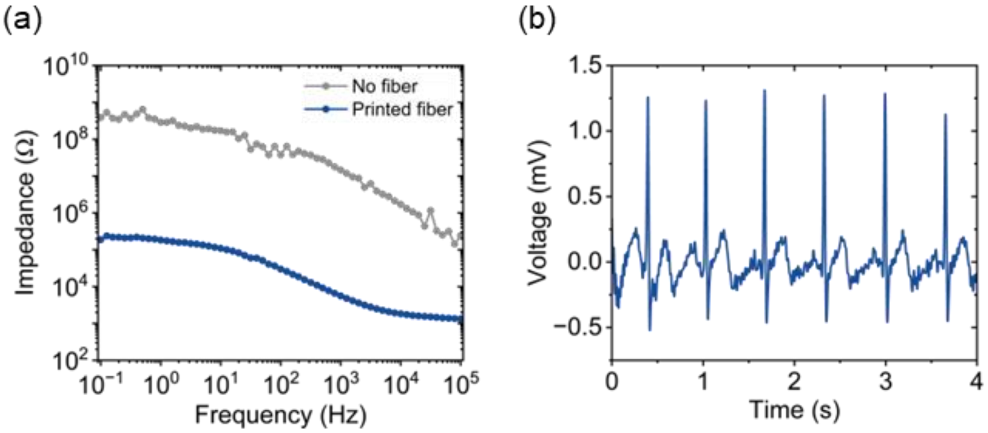

Figure S14. PEDOT:PSS microfiber electrode to enable biopotential measurements from a plier handle. Contact impedance (a) and ECG signal (b) measured through the handle of a plier with printed PEDOT:PSS fiber arrays.

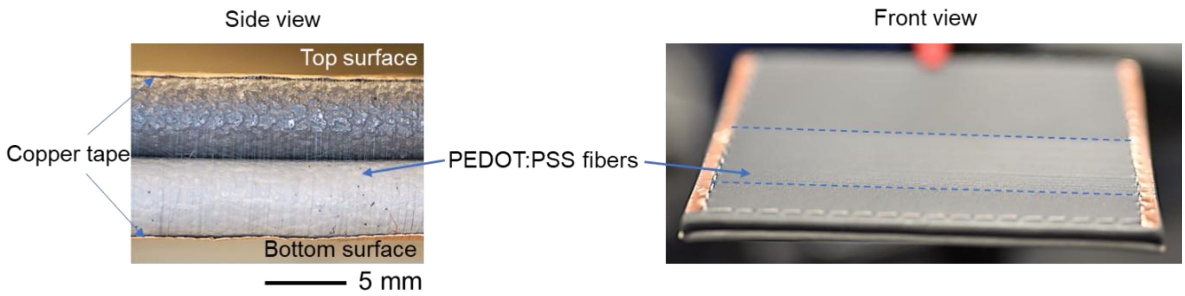

Figure S15. PEDOT:PSS microfibers deposited on a coaster. A side view and a front view photos of the coaster, on which the PEDOT:PSS fibers are printed to connect the top and bottom surfaces of this coaster.

a)

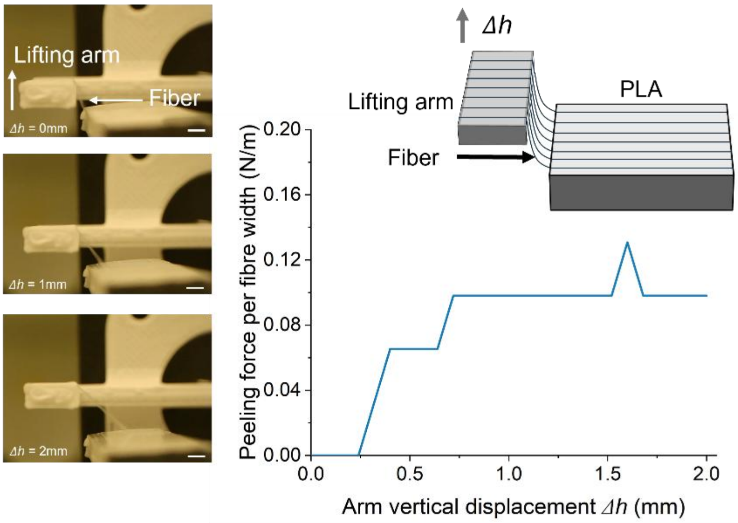

b)

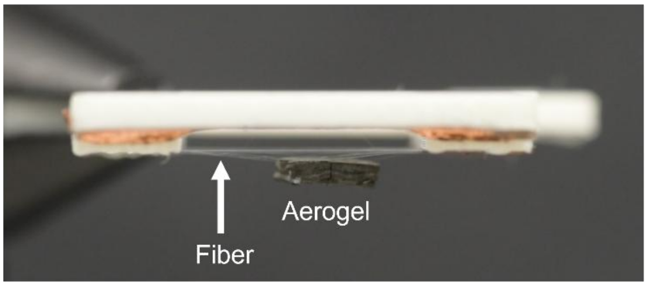

Figure S16. Adhesion of PEDOT:PSS microfibers on object surface. (a) In the process of a modified 90° peeling test (derived from ASTM D2861 90-degree peeling test), the microfiber array (N/d ~ 200/10mm) is printed onto the PLA part and a lifting arm. The peeling force per unit fiber width could be measured by $F = \frac{W}{dN}$, where W is the reaction measured when the lifting arm is peeling the fiber (the PLA part is placed on a digital mass balance), d is the fiber diameter, and N is the fiber number. (b) A photo showing a layer of fiber array is able to suspend the aerogel in the air.

References

1. Wang W, Pan Y, Shui Y, Hasan T, Lei IM, Ka SGS, Savin T, Velasco-Bosom S, Cao Y, McLaren SBP, Cao Y, Xiong F, Malliaras GG, Huang YYS. Imperceptible augmentation of living systems with organic bioelectronic fibres. Nat Electron. 2024;7:586–97.
